# On the role of steric clashes in methylation control of restriction endonuclease activity

**DOI:** 10.1093/nar/gkv1341

**Published:** 2015-12-03

**Authors:** Karolina Mierzejewska, Matthias Bochtler, Honorata Czapinska

**Affiliations:** 1 International Institute of Molecular and Cell Biology, Trojdena 4, 02-109 Warsaw, Poland; 2 Institute of Biochemistry and Biophysics PAS, Pawinskiego 5a, 02-106 Warsaw, Poland

## Abstract

Restriction-modification systems digest non-methylated invading DNA, while protecting host DNA against the endonuclease activity by methylation. It is widely believed that the methylated DNA would not ‘fit’ into the binding site of the endonuclease in the productive orientation, and thus steric clashes should account for most of the protection. We test this concept statistically by grafting methyl groups *in silico* onto non-methylated DNA in co-crystal structures with restriction endonucleases. Clash scores are significantly higher for protective than non-protective methylation (*P* < 0.05% according to the Wilcoxon rank sum test). Structural data alone are sufficient to distinguish between protective and non-protective DNA methylation with 90% confidence and decision thresholds of 1.1 Å and 48 Å^3^ for the most severe distance-based and cumulative volume-based clash with the protein, respectively (0.1 Å was deducted from each interatomic distance to allow for coordinate errors). The most severe clashes are more pronounced for protective methyl groups attached to the nitrogen atoms (N6-methyladenines and N4-methylcytosines) than for C5-methyl groups on cytosines. Cumulative clashes are comparable for all three types of protective methylation.

## INTRODUCTION

Restriction-modification (RM) systems consist of endonucleases that cleave invading unmodified DNA and their cognate methyltransferases of matching or slightly broader specificity, which protect genomic DNA of the host from this fate. Hemimethylated DNA arises after semi-conservative replication of fully methylated DNA and is the preferred substrate for the RM methyltransferases. *In vitro* they also modify non-methylated DNA which tends to blur the distinction between host and invading DNA. In order to avoid damage to the host genome during DNA replication, hemimethylation is typically sufficient to prevent restrictase-mediated DNA cleavage. The effects of non-cognate methylation (of the wrong type, in the wrong place, introduced chemically or by a methyltransferase from a different RM system) range from full to no protection. Experimental data on the influence of methyl groups on the susceptibility of DNA to cleavage by restriction endonucleases have been collected in the REBASE database (http://rebase.neb.com) (1).

Crystallographic studies directly addressing the interaction of restriction endonucleases with modified DNA are scarce, because cognate methylation prevents productive (and tight) binding of DNA and therefore interferes with crystal formation. Although direct evidence is limited, it is widely assumed that methyl groups inhibit DNA cleavage simply because they do not fit into the binding clefts of the cognate restriction enzymes in a reaction compatible way. The concept is plausible, because steric clashes are associated with high penalties (2). However, adaptive fit between protein and DNA may significantly reduce the contribution of steric clash penalties to the distinction between methylated and non-methylated DNA.

Here we statistically address the role of steric clashes in the control of restriction endonuclease activity by DNA methylation. Such analysis has become possible thanks to the rise in the number of crystal structures of restriction endonucleases with DNA. Currently, altogether 174 structures of 51 restriction enzymes have been solved. Among these, 111 structures capture type II restriction endonucleases bound to cognate DNA in a substrate- or product-like orientation. All show the enzymes bound to non-methylated DNA (modification dependent IIM endonucleases, e.g. DpnI, AbaSI or MspJI were excluded from this study). The methyl group positions can be directly deduced from the coordinates of the DNA bases. The C5 methyl group on cytosine is located in the plane of the base as a direct consequence of its aromaticity. The cytosine N4 and adenine N6 methyl groups are driven into this plane by conjugation of the nitrogen electrons with the aromatic ring and out of it by short intramolecular contacts. The conjugation effect prevails and the methyl groups are located within about 10° (or 0.25 Å) of the base plane (3). The two methyl groups can be located either on the Watson-Crick (*cis* form) or Hoogsteen side of the base (*trans* form) (4). Although in the context of isolated base the *cis* conformation prevails (5), due to base pairing it is disfavored in double-stranded DNA and the *trans* form is observed instead (6,7). Hence the location of the N4 and N6 methyl groups is also inferable from the coordinates of the base alone.

**Figure 1.**
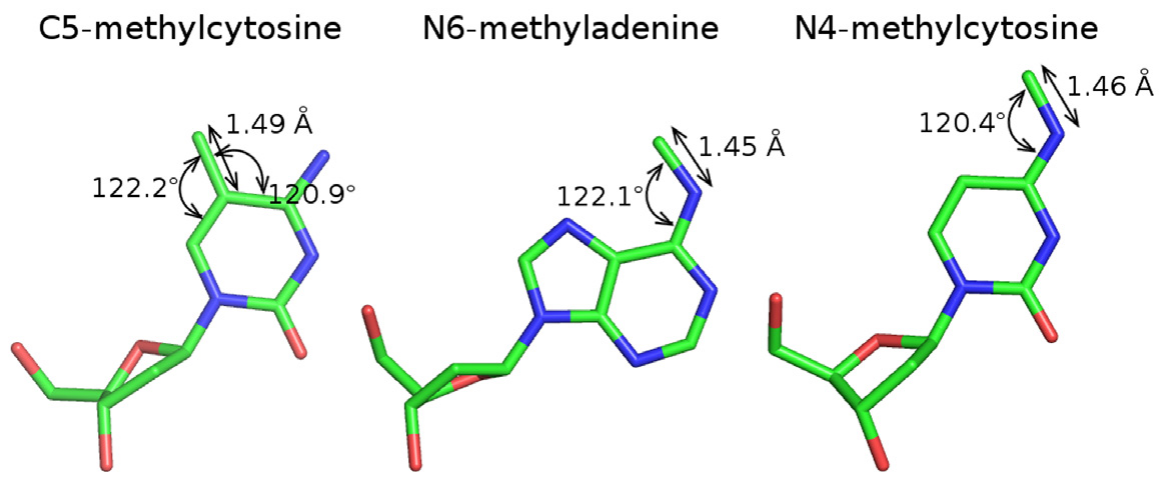
Selected geometric parameters of the methylated bases used for the deduction of the methyl group positions. Only the base atoms were used for superposition with the non-methylated bases present in the restriction endonuclease-DNA crystal structures, sugars are shown for clarity.

We grafted methyl groups (with implicit hydrogen atoms) on non-methylated DNA in co-crystal structures with proteins (for detailed bond lengths and angles, see Figure 1). We analyzed three types of cases: the methylation introduced by cognate methyltransferase of an RM system, the non-cognate methylation that interfered with DNA cleavage and the non-cognate methylation that did not affect the endonuclease activity.

## MATERIALS AND METHODS

### Input data

The set of 30 structures of restriction endonuclease–DNA complexes was downloaded from the Protein Data Bank (3)(Supplementary Table S1). The resolution of the crystal structures used for this study ranged between 1.3 Å and 3.2 Å, with a median of 2.0 Å. As hydrogen atoms are not resolved at this resolution, they were treated implicitly. Cruickshank's estimates (8) show that most coordinate errors should be below 0.1 Å. An error of 0.1 Å was therefore assumed for all interatomic distances.

### *In silico* methylation

The methyl group positions were modeled based on the superposition of methylated bases onto the non-methylated ones originally present in the restriction endonuclease–DNA complex structures. The structures of: mouse ZFP57 zinc finger DNA complex (9), restriction endonuclease DpnI DNA complex (10) and synthetic hexanucleotide d(CGCGm4CG) (11), served as sources for the 5-methylcytosine (m5C), N6-methyladenine (m6A) and N4-methylcytosine (m4C) coordinates, respectively. The methyl groups of m6A and m4C were rotated so that they lie precisely in the plane of the base. The m6A base was additionally adjusted to match the stereochemical parameters of the small molecule structure (4). The geometric parameters for the location of the methyl groups in the template structures are presented in Figure 1.

### Van der Waals radii of atoms

The van der Waals radii were assigned with hydrogens atoms treated implicitly based on values from Richards (12). For example, for the oxygen atoms of the COOH group we used the radii of 1.5 Å, as a compromise between the values for carbonyl and hydroxyl groups. Similarly, in the asparagine and glutamine residues, the carbonyl and amino parts of the CONH_2_ group were assigned an averaged value of 1.6 Å. Since we did not analyze the geometry of the clash, we did not treat the ring atoms of phenylalanine, histidine, tyrosine and tryptophan and guanidino group of arginine as ellipsoids with larger dimension in the plane of the aromatic systems, but we assumed a ball approximation with the smaller (1.7 Å) radius instead.

To our knowledge there were no detailed studies concerning van der Waals radii of DNA atoms. Therefore we deduced them based on analogy to protein data. We treated the ring atoms of the bases similarly as protein aromatic rings (1.7 Å in all dimensions), oxygen atoms of C, T and G bases as the protein main chain oxygen (1.4 Å), the NH_2_ group of C, A and G bases analogously to the guanidino group of Arg (1.7 Å). The methyl group of T and the *in silico* introduced methyl groups were treated as standard aliphatic methyl groups (2.0 Å). The oxygen atoms in sugar and phosphate groups were assigned a value of 1.4 Å analogous to the carbonyl oxygen atom of proteins (oxygen atom without hydrogens). The radius of the phosphate atom (1.8 Å) was based on the work of Bondi (13). All van der Waals radii used in this study are presented in Table 1.

**Table 1.** The list of van der Waals radii used in this study

Atom/group type	Residue/base/group	Van der Waals radius [Å]
**Protein**		
C (carbonyl) or NH	main chain	1.7
O	main chain	1.4
Cα	main chain	2.0
C	COOH, CONH2	1.7
O	COOH	1.5
O, N	CONH2	1.6
ring atom	His, Phe, Tyr, Trp	1.7
C, NH, NH2	guanidino group of Arg	1.7
OH	Ser, Thr, Tyr	1.6
CH,CH2,or CH3	aliphatic	2.0
NH2	Lys	2.0
S	Met, Cys	1.8
all other protein atoms		2.0

**DNA**		
ring atom	all bases	1.7
exocyclic NH2	Ade, Gua, Cyt	1.7
exocyclic O	Gua, Cyt, Thy	1.4
exocyclic CH3	Thy	2.0
O	sugar, phosphate	1.4
P	phosphate	1.8
all other DNA atoms		2.0

### Definition of clashes and the clash score

A clash was defined as a pair of (non-hydrogen) atoms coming closer to each other than the sum of their van der Waals radii, reduced by 0.1 Å to take into account coordinate uncertainties. Thus, the distance based clash was calculated according to the formula:

\begin{equation*} \Delta d = R + r - d \end{equation*}

where *R* is a van der Waals radius of methyl group (2 Å), *r*—van der Waals radius of another atom/group (see Table 1), *D*—distance between the methyl group and the other atom/group and *d = D + 0.1 Å*—distance taking into account the approximate 0.1 Å uncertainty of crystallographic coordinates. The volume based clash was calculated based on the equation for overlapping spheres (14):

\begin{eqnarray*} &&\Delta V= \nonumber \\ &&(\pi (R{+} r{-} d)^2 (d^2 {+} 2dr {-} 3r^2 {+} 2dR {+} 6rR {-} 3R^2 ))/12d \end{eqnarray*}

We have next calculated cumulative clashes for each methyl group in both distance and volume domain. Finally we have determined the number of contacts and contacted residues. The obtained results are presented in Supplementary Table S2.

### Statistical analysis

Data were partitioned into a test set with all instances of ‘biologically relevant’, protective methylation (by a cognate methyltransferase of fully characterized specificity), a reference set of all instances of ‘biologically irrelevant’ non-protective DNA methylation (either by an unrelated methyltransferase or chemical) and a ‘validation’ set of non-biological protective methylation (Supplementary Table S2 and Figure S1). The statistical analysis was performed using Scipy package for Python, v 0.15.1. As most clash scores are not normally distributed according to the Shapiro–Wilk test for normality (H0 = data drawn from normal distribution) (15,16), Wilcoxon rank sum tests (two-sided, H0 = two sets of measurements drawn from the same distribution) were used for comparisons (Supplementary Table S3). Finally, the robustness of the results with respect to coordinate uncertainty and adaptive fit was verified by incremental increase of the 0.1 Å error allowance (Supplementary Table S4).

## RESULTS AND DISCUSSION

### Dataset

Structures of type II restriction endonucleases with DNA bound in a productive orientation (excluding the type IIM enzymes) were obtained from the Protein Data Bank (PDB) (3). Equal statistical weight was given to enzymes, not to PDB-submissions or molecules in the asymmetric unit to avoid bias from the focus on a few restriction enzymes selected as ‘prototypical’ models. When multiple PDB instances were available for a particular endonuclease, we chose the ‘best’ structure (according to resolution, lack of experimentally introduced mutations, etc.). In the case of multiple copies in the asymmetric unit we averaged the results. The accession codes of PDB structures used in this work are listed in Supplementary Table S1. Altogether 30 different endonucleases were studied, among these, 8 belong to RM systems with C5-methyltransferase (for 3 of them it is not known which cytosine is methylated), 7 to RMs with adenine N6-methyltransferase and 14 to RMs with N4-methyltransferase (for 5 of them it is not known which cytosine is methylated). One enzyme, PacI, does not have a cognate methyltransferase, the protection of the host genome is in this case ensured by the absence of the endonuclease target sequence (17).

### *In silico* methyl group grafting and clash evaluation

Methyl groups were computationally grafted onto the DNA, in the plane of the bases and in the *trans* form in case of N4 and N6 methylation (on the Hoogsteen side). Next we have analyzed the environments of the introduced modifications. All contacts between the methyl and the rest of the endonuclease–DNA complex that were shorter than the sum of van der Waals radii of interacting atoms were marked as steric conflicts. In addition to the distance based clashes, we also calculated the overlap of the corresponding van der Waals spheres (a weighted clash measure ensuring that the most severe clashes have more impact than the minor ones) and the number of contacted atoms/residues (Supplementary Table S2).

**Figure 2.**
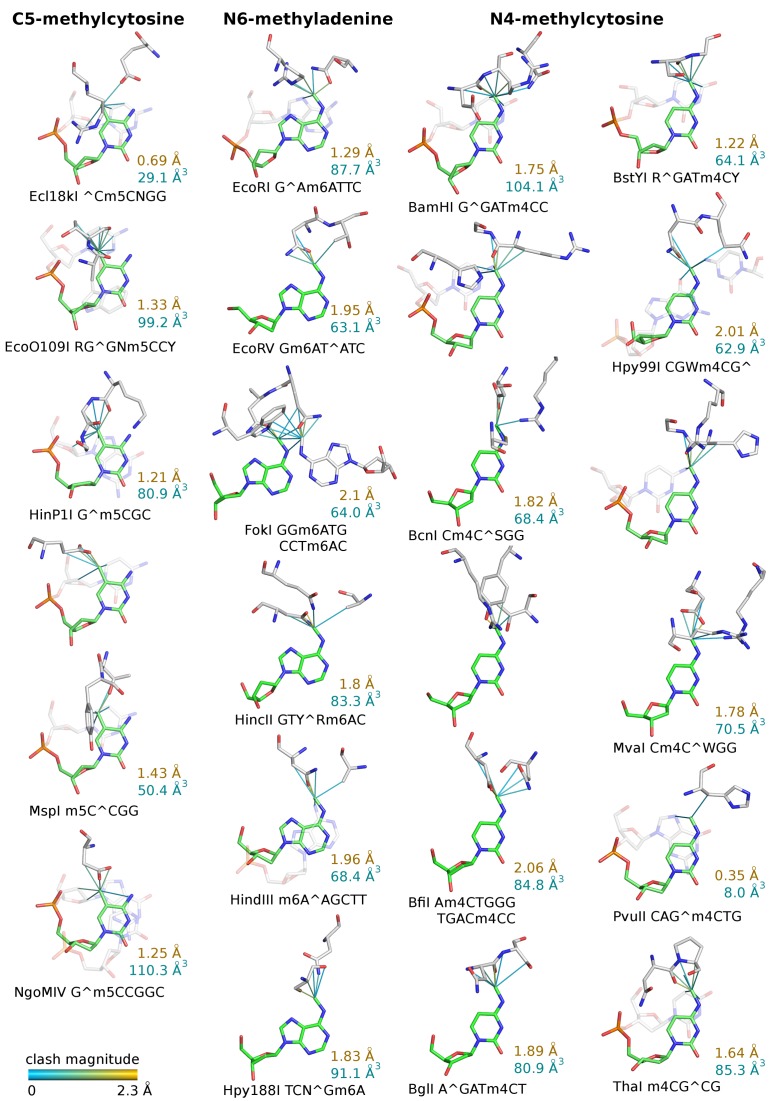
Clashes of protective methyl groups introduced by cognate methyltransferases with the protein and DNA atoms in the restriction endonuclease–DNA complex structures. The clashes are colored according to their magnitude (from cyan to yellow for least to most severe overlap). The MunI structure has been omitted due to high similarity to EcoRI endonuclease, particularly pronounced in the methyl group environment. The position and type of cognate methylation is indicated in restriction enzyme target sequences (^∧^ marks the cleavage site). The magnitude of the most severe clash and the cumulative volume clash is shown in yellow and cyan, respectively.

**Figure 3.**
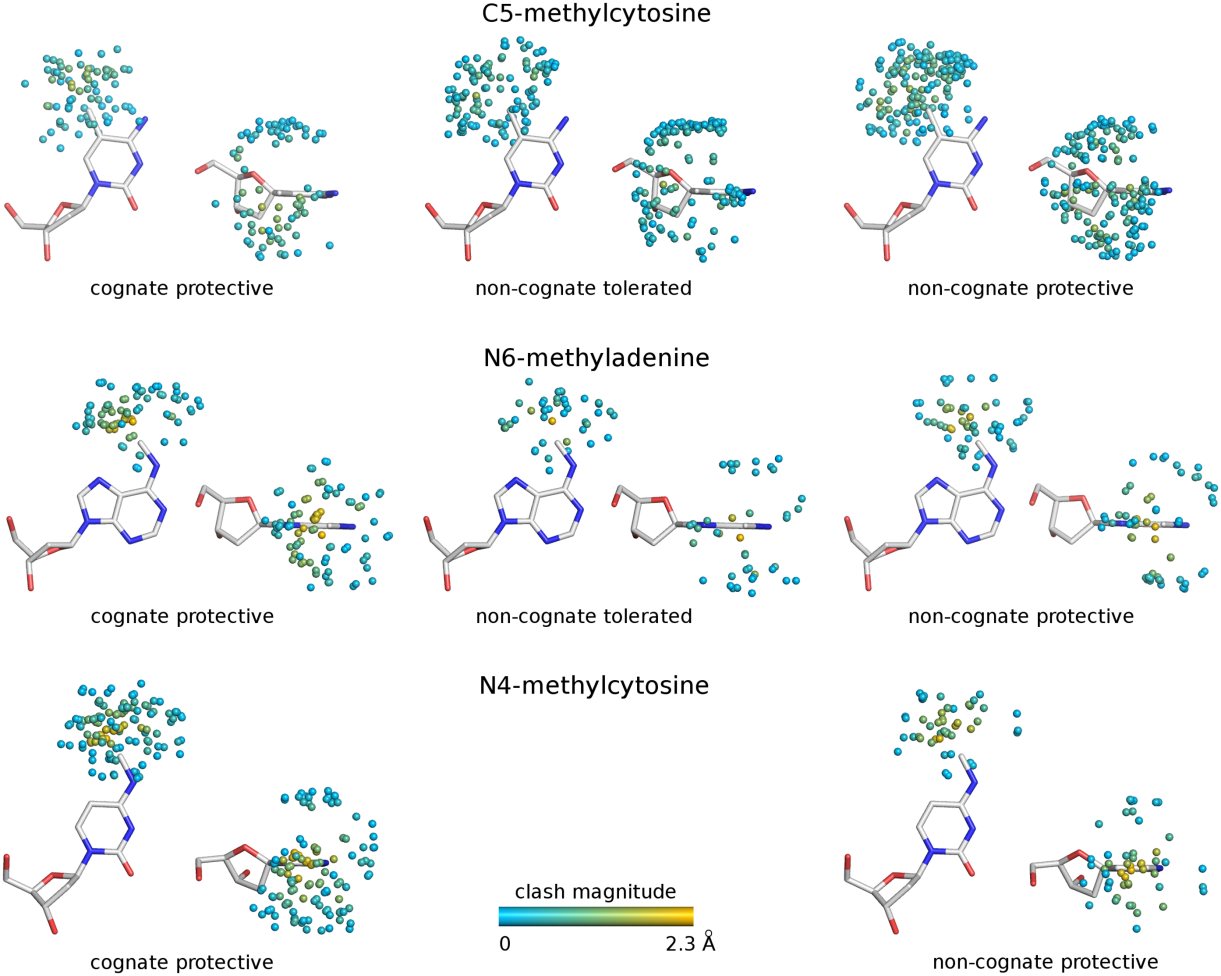
Spatial distribution of atoms potentially clashing with the methyl groups. Only the atoms for which the distance to the methyl group is below the sum of the van der Waals radii (allowing 0.1 Å for coordinate uncertainty) are shown and colored according to the severity of the steric conflict. Only one molecule in the asymmetric unit was chosen. The clashes of the methyl groups on both DNA strands are included.

First, the environments of the methyl groups introduced by cognate methyltransferases were analyzed. Most of the methyl groups clashed with restriction endonuclease atoms, as expected. The detailed steric conflicts for cognate methylation are illustrated in Figure 2. Next, the analysis was repeated for the methyl groups, which did not protect against DNA cleavage (even when the methyl group was present in both strands). Many of the non-protective methyl groups were found to clash as well, but steric conflicts tended to be less severe than for the cognate ones (Figure 3, left and middle columns). Finally we examined the effect of non-cognate, but protective methylation, only taking into account cases where methylation of one strand was already protective to mimic the situation observed for the RM systems. The clash distribution of non-cognate protective methyl groups resembled the one observed for cognate methylation (Figure 3, left and right columns).

### Most severe clashes differ between protective and non-protective methyl groups

**Figure 4.**
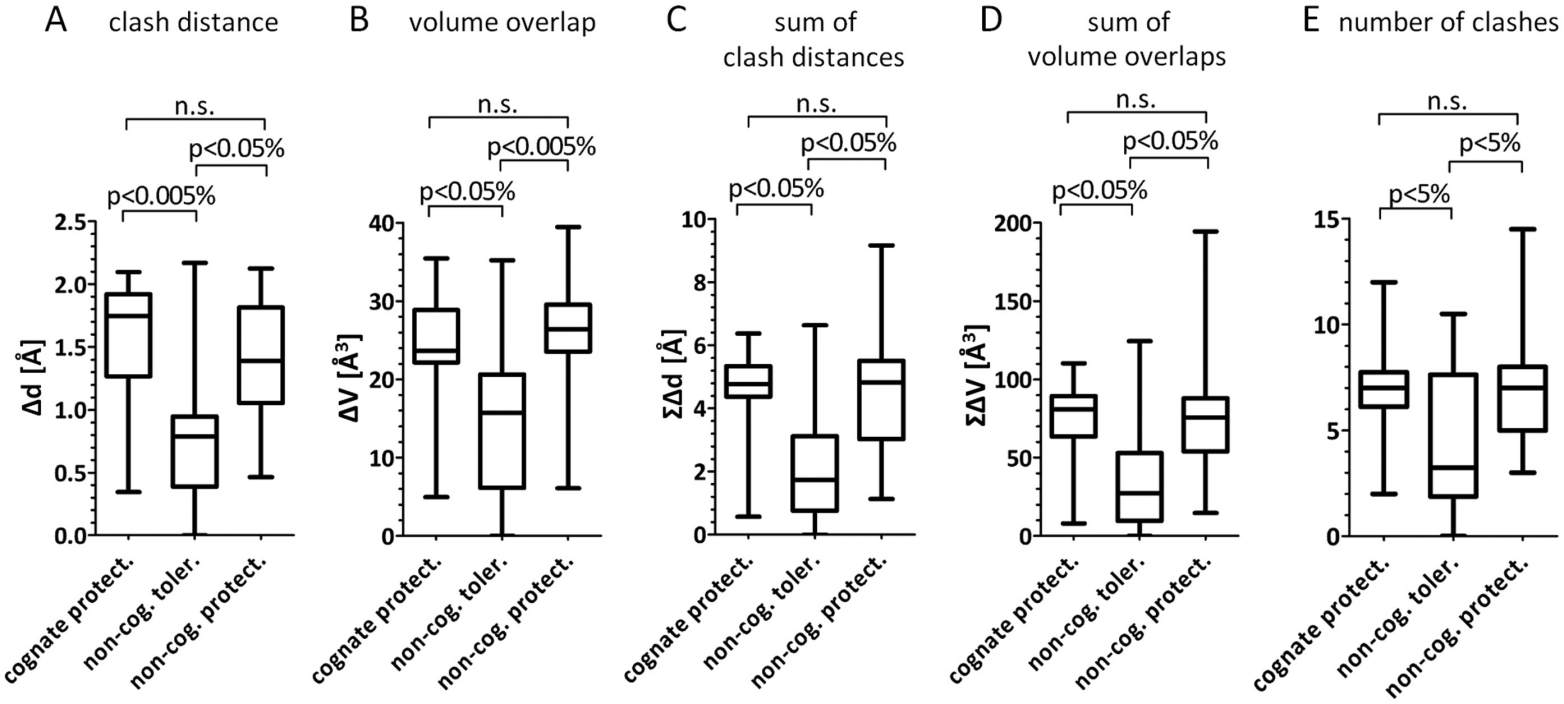
Analysis of the severity of clashes in case of cognate protective, non-cognate tolerated and non-cognate protective methylation. *P*-values are based on Wilcoxon rank sum test; n.s.—non-significant difference. Whiskers show min and max values. Horizontal bars represent median.

The energetic penalty for the introduction of a methyl group should be dominated by its most severe steric conflict (2), at least when the protein and DNA are treated as rigid entities. Therefore we first scored only the largest distance and volume based clashes for each methyl group and then compared these for the three sets of methyl groups, using the Wilcoxon rank sum test (Figure 4A and B). In both cases, the protective methylations differed significantly from the non-protective methylation (*P*-values below 0.05%). For all analyzed enzymes (known type and position of biological methylation), the two most severely clashing methyl groups separately either convey protection against nuclease cleavage or have unknown effect (Supplementary Figure S2 and Table S5). Clashes of cognate and non-cognate protective methyl groups were of similar magnitude, arguing against extensive evolutionary optimization of restriction endonucleases for maximum steric conflict with DNA that has been methylated by the associated methyltransferase. Therefore, clash score based predictions of the type and position of cognate methylation are correct in less than half of all analyzed cases only (Supplementary Figure S2 and Table S5).

### Cumulative clash scores differ between protective and non-protective methyl groups

Taking adaptive fit into account, penalties for steric conflicts may not be dominated by the most severe ones. Instead, a larger number of minor clashes might be harder to relieve than a single major one. In order to test this idea, we have calculated the sums of distance and volume based clashes and compared them using the Wilcoxon rank sum test (Figure 4C and D). The differences in clash scores for protective and non-protective DNA methylation were of similar significance (*P*-values below 0.05%), indicating that in addition to the most severe ones, other conflicts contribute as well (otherwise the distinction should get worse by the inclusion of ‘noise’). On average, the most serious clash contributed about a third of the total overlap volume. As before, there was no strong evidence that clashes with methyl groups that are installed by the cognate methyltransferases are more severe than the ones that are not biologically relevant, but protect DNA *in vitro* (Figure 4CD). Finally, we compared the number of steric conflicts for the three groups of modifications. This score gives undue weight to very minor clashes and as expected is less suitable to distinguish between protective and non-protective methylation (Figure 4E).

### Increase of the distance uncertainty does not significantly affect the clash scores

We tested whether the discriminative power of clash measures could be improved if the 0.1 Å allowance for the coordinate errors was enlarged to accommodate adaptive fit effects. An increase of the allowed range should reduce the magnitude of drastic clashes to a limited extent and eliminate the small clashes that could easily be relieved by minor adjustment of the protein and/or DNA interface. On the other hand, too much allowance for adaptive fit should lead to the omission of clashes that contribute to methyl-sensitivity. Wilcoxon rank sum tests showed that a gradual increase of the uncertainty in the interatomic distances from 0.1 to 0.4 Å had a very minor effect on the statistical significance of the results. Moreover, no clear trend could be observed and instead various measures behaved differently in the analysis (Supplementary Figure S3 and Table S4). We conclude from this data that the discriminative power of clash scores is relatively robust with respect to small coordinate offsets.

### Clashes with protein main chain and side chains

**Figure 5.**
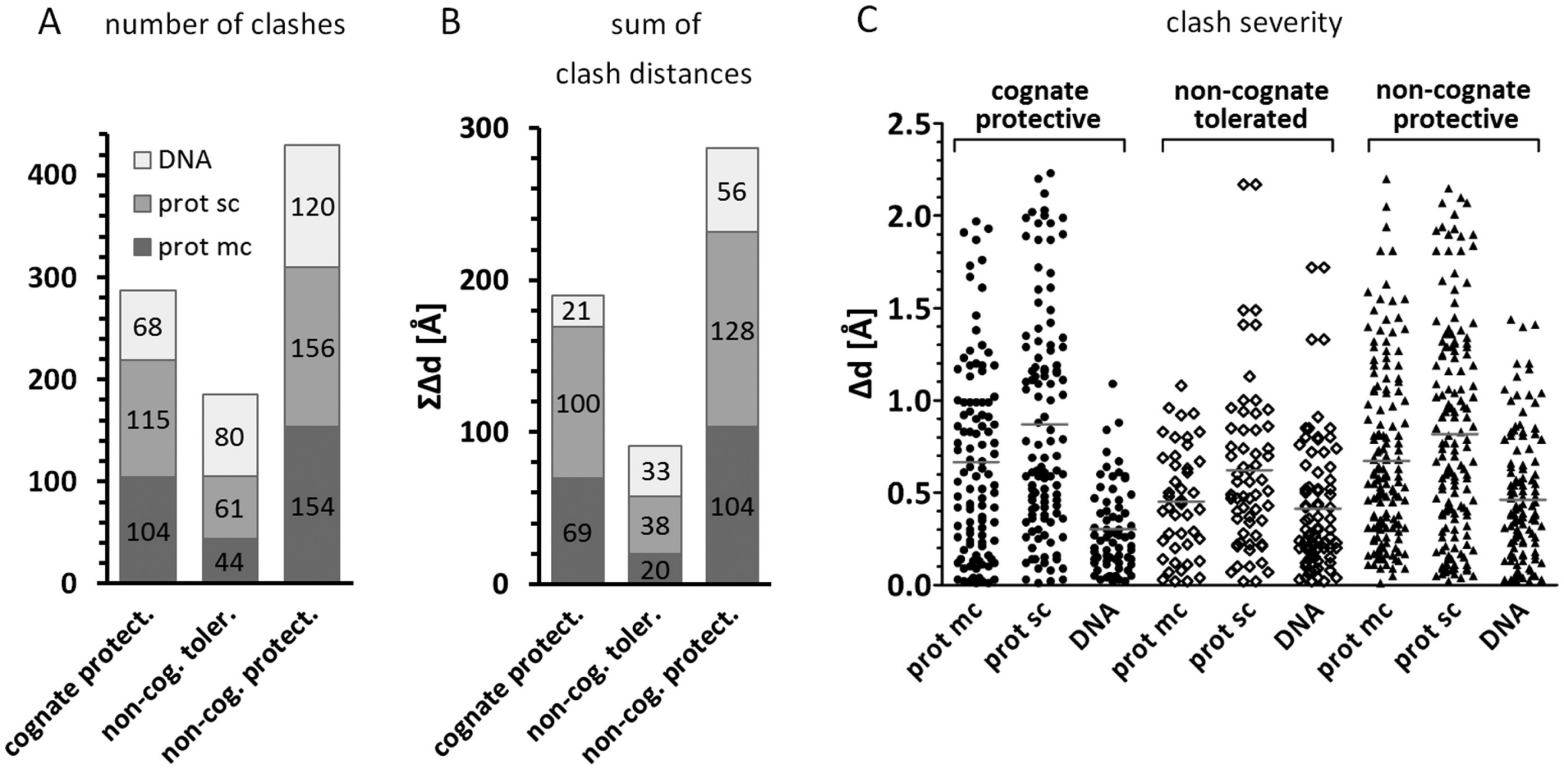
Distance based clashes divided according to the type of atom contacting the methyl group. For each enzyme one representative structure was chosen and methyl groups of the two DNA strands were analyzed independently. In case of multiple molecules in the asymmetric unit, only one was chosen. (**A**) number of clashes; (**B**) sum of clashes; (**C**) clash severity, each dot represents one clash and horizontal lines represent mean. Prot sc—protein side chain, prot mc—protein main chain.

Steric conflicts of grafted methyl groups may affect DNA binding in several different ways. Methyl groups could clash with either protein backbone or its side chains. Due to the fact that the Cβ atom position is strictly defined by the protein backbone we have included it in the main chain atom set. Clashes with the protein backbone and its side chains were more numerous (Figure 5A), more severe in total (Figure 5B) and more drastic (Figure 5C) for protective than for non-protective DNA methylation. Side chain atoms may be more flexible and thus easier to ‘move out of the way’ than the protein backbone, nonetheless for both protective and non-protective methylation, they are slightly more abundant and pronounced (Figure 5 and Supplementary Figure S1). When the clashes with the side chains were omitted, the *P*-values of the Wilcoxon rank sum test were much higher (worse) for all clash measures that we used to compare protective and non-protective methylation. This might reflect the better access of side chains (compared to the backbone) to methyl groups in the major groove.

### Clashes with DNA

The methyl groups may also clash with other DNA atoms of either the same or the opposite strand. Surprisingly, steric conflicts of grafted methyl groups with DNA were slightly more numerous and severe for non-protective than protective methylation, but the difference was not significant according to Wilcoxon rank sum tests (*P* > 5%) (Figure 5 and Supplementary Figure S1). As this small effect may be due to a few outliers, we concluded that methyl groups modulate DNA susceptibility to restriction endonuclease cleavage by direct interactions with the protein rather than by intra-DNA steric clashes (which might deform DNA and thus indirectly influence protein binding). Our conclusions were reinforced by the observation that omission of the clashes with DNA had a positive effect on Wilcoxon rank sum scores (Supplementary Table S3).

The absence of clashes between symmetrically introduced methyl groups is remarkable. The dataset includes enzymes (EcoRI and FokI) associated with methyltransferases that modify adenines in the palindromic AT context favoring methyl-methyl clashes (see Mierzejewska *et al*., Supplementary Table S3 (18)). Nevertheless, it seems that none of the two enzymes exploits constraints imposed by methyl group proximity on DNA structure. Only a minor clash between the two methyl groups is observed for FokI endonuclease (0.27 Å) and none at all for EcoRI that kinks the DNA in between of the two AT pairs so that the methyl groups are 7.6 Å from each other. The absence of RM systems using methyl-methyl clash to prevent DNA cleavage contrasts starkly with the exploitation of the same effect for licensing of the reaction by methyl dependent DpnI endonuclease (18). This may reflect the biological need for methylation to afford not only protection of fully methylated, but also hemimethylated DNA.

### Comparison of C5, N6 and N4 methyl group clashes

**Figure 6.**
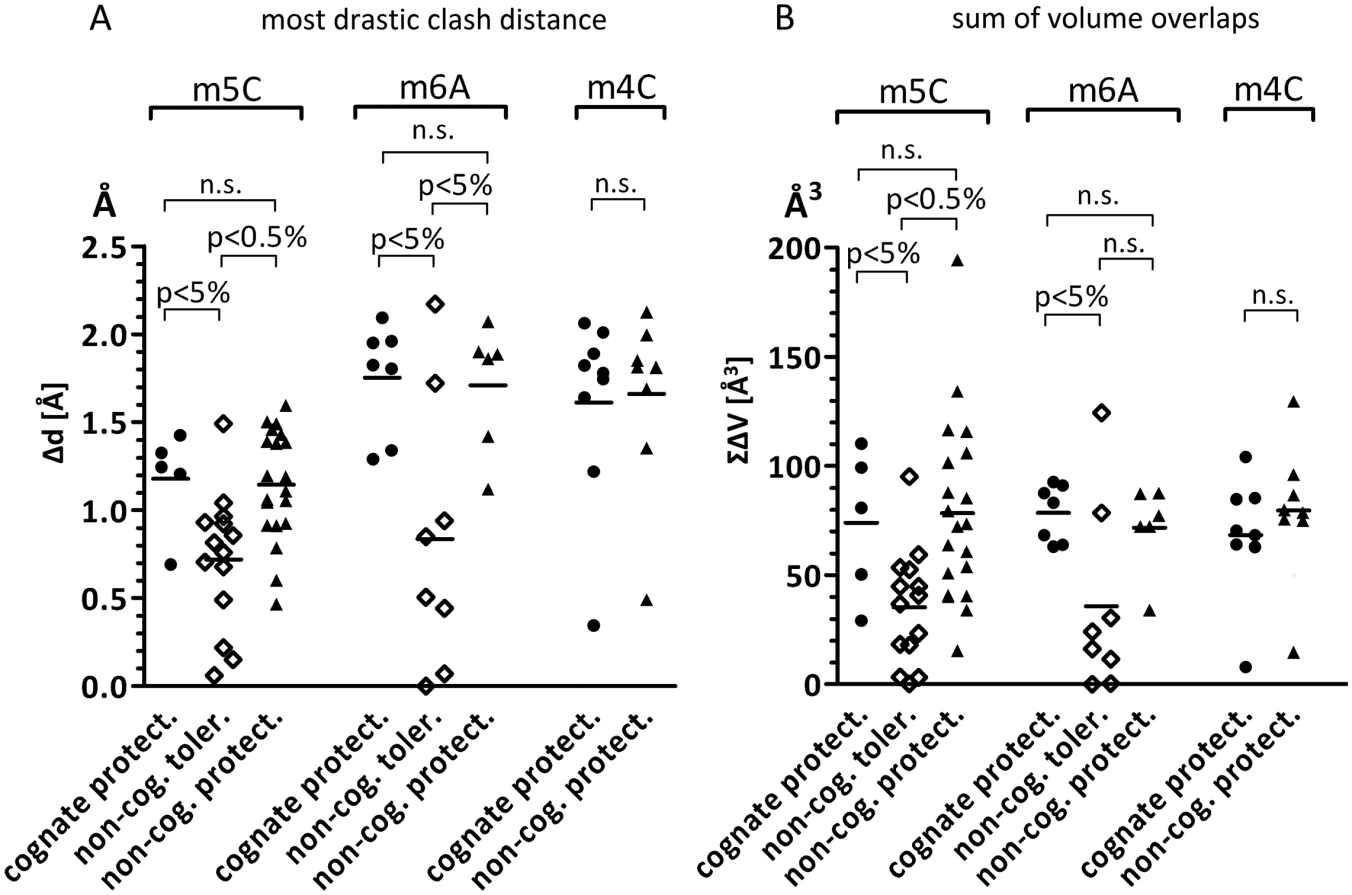
Severity of clashes analyzed according to the type of methylation. Each dot corresponds to one enzyme, for which a representative structure was chosen and methyl groups from both strands as well as from multiple molecules in the asymmetric unit were averaged. (**A**) most drastic distance based clash; (**B**) sum of overlapping volumes. *P*-values are based on Wilcoxon rank sum test; n.s.—non-significant difference. Horizontal lines represent mean.

To have a largest possible dataset and best statistics in the above study we have pooled together all types of methylation (C5 cytosine, N6 adenine and N4 cytosine). However, the three methyl groups may behave differently and thus we repeated the above analysis treating them separately. We found 5/14/20, 7/8/6 and 9/0/9 instances of C5 cytosine, N6 adenine and N4 cytosine cognate/tolerated/non-cognate protective methylation, respectively. No double-stranded tolerated N4 methylation was reported in the REBASE database and thus it could only be analyzed indirectly in relation to somewhat similar N6 methylation. For C5 and N6 methylation, the numbers were also relatively small, but sufficient for a statistical comparison of clash scores. For all types of methylation, the above conclusions remained valid and protective methyl groups clashed more severely than tolerated ones (Figure 6A and Supplementary Table S3B–D). However, both cognate and non-cognate protective methylations were associated with more substantial steric conflicts for adenine N6 and cytosine N4 than for cytosine C5 methyl groups.

**Figure 7.**
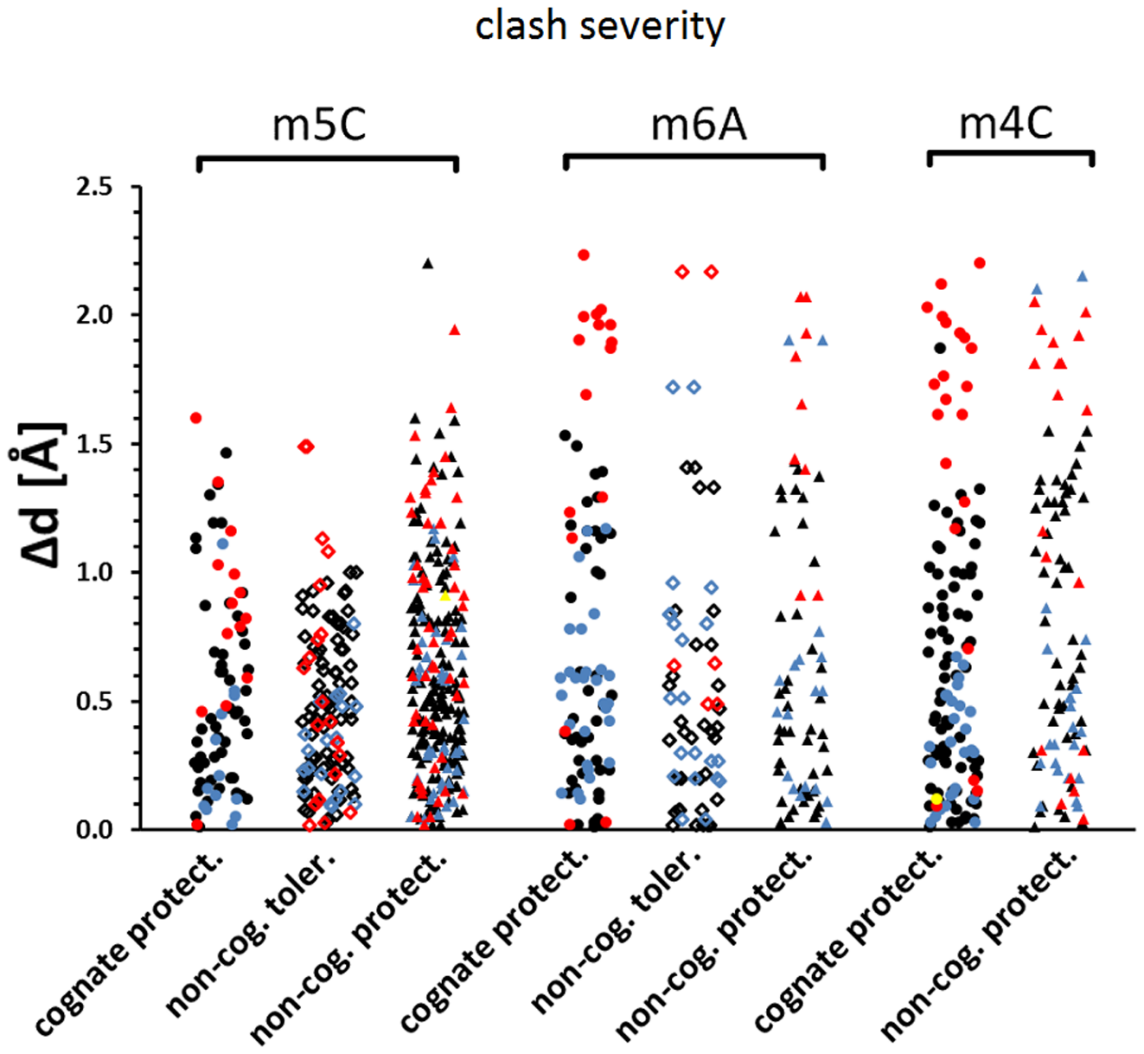
Steric conflicts of all methylation types analyzed according to the type of clashing atoms. For each enzyme one representative structure was chosen and methyl groups from both DNA strands were analyzed independently. In case of multiple molecules in the asymmetric unit, only one was chosen. Each dot corresponds to one clash. Clashes with carbon, nitrogen, oxygen and sulfur were colored in black, blue, red and yellow, respectively.

We considered several possible explanations for this effect. First, C5 methyl groups are close to the backbone phosphates, and less accessible than N4 and N6 methyl groups. Therefore, it may be easier to introduce severe steric conflict with an exocyclic nitrogen attached methyl group than with a C5 methyl group, when clashes with other DNA atoms are to be avoided to keep non-methylated DNA bound and cleaved. Second, the extent of methyl group clashes may depend on the environment of the heteroatom to which the methyl group is attached. The exocyclic amino groups of adenine and cytosine act as hydrogen bond donors and hence one may expect a hydrogen bond acceptor in hydrogen bonding distance (2.5–3.4 Å) (19) in the absence of methylation. In contrast, the C5 atom of cytosine cannot engage in such interactions, and hence endonuclease (non-hydrogen) atoms should be at least 3.5 Å away from the C5 atom itself. If clash scores were significantly influenced by interactions of the non-methylated DNA with restriction endonucleases, then most clashes of N6 and N4 methyl groups should be with hydrogen bond acceptors, which in proteins are typically represented by oxygen atoms. An analysis of clashes according to atom type confirmed that indeed most severe clashes with N6 and N4 methyl groups are caused by oxygen atoms, which is not the case for C5 methyl groups (Figure 7 and Supplementary Figure S4).

N6 and N4 methyl groups should also be easier to rotate out of the plane of the base than C5 methyl groups. In other words, not only maximal but also cumulative clashes should be more severe to be protective in the first two cases. The cumulative clash analysis does not support this hypothesis (no significant difference is observed between the three methylation types, Figure 6B and Supplementary Table S3). Instead, it suggests that an overall ‘crowded’ environment compensates for less severe maximal clashes in the case of C5-methylcytosine.

### Clash scores as predictors of protective and tolerated methylation

The statistical measures presented above provide clear evidence that repulsive van der Waals interactions play an important role in methylation-mediated DNA protection against endonuclease cleavage. However, they depend on the sample size (the *P*-values are expected to get lower as more structures are being solved) and are therefore unsuitable to tell whether clash scores can distinguish between protective and tolerated DNA methylation in any given instance.

**Figure 8.**
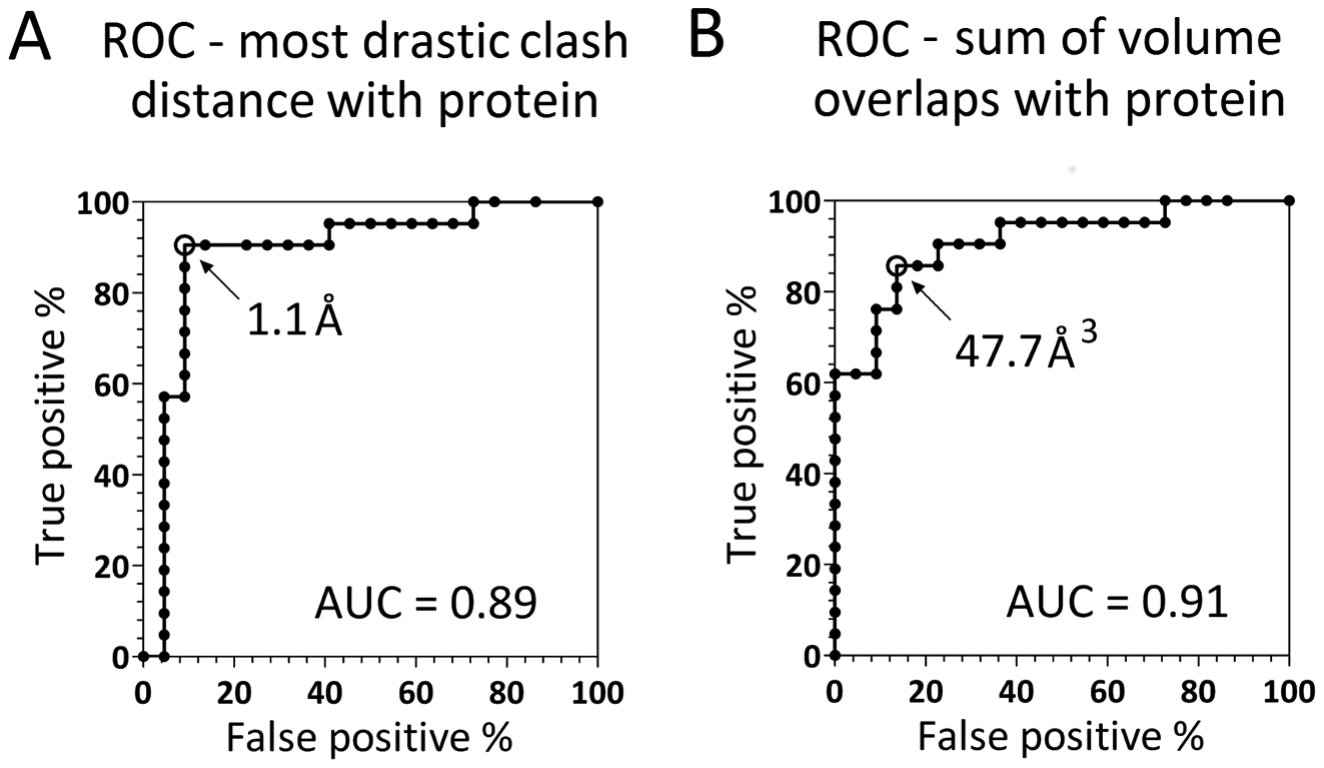
Receiver Operating Characteristic (ROC) curves for the two best predictors of protective methylation. (**A**) Most drastic distance based clash of the methyl group and the protein. The study predicts with 90 and 9% of true and false positives, respectively, that a methyl group protects from endonuclease cleavage if it is more than 1.1 Å closer to a protein atom than the sum of the van der Waals radii (reduced by 0.1 Å to account for coordinate errors). (**B**) Cumulative volume overlap between the methyl group and the protein atoms. The analysis predicts with 86 and 14% of true and false positives, respectively, that a methylation is protective if it is overlapping more than 47.7 Å^3^ in volume with all protein atoms.

In order to better understand the predictive value of the steric conflict, we pooled cognate and tolerated methyl groups and predicted protection against endonuclease cleavage (treated as a ‘positive’) in those cases where the clash score exceeded a threshold. To avoid setting an arbitrary threshold, we calculated the receiver operating characteristics (ROCs). In ROCs the decision threshold is treated as a parameter and the rate of true positives is measured as a function of the rate of false positives. The area under the ROC curve (AUC) can then serve as a measure of the predictor quality. For a perfect predictor it should be 1, for a random predictor 0.5. As several clash scores (maximum/sum of distances/volumes) performed similarly in the Wilcoxon rank sum tests, we tested the different measures separately (Figure 8 and Supplementary Figure S5). The area under the curve (AUC) parameter agreed with the Wilcoxon rank sum test *P*-values.

The cumulative volume overlap between the methyl groups and protein atoms was found to be the parameter with the highest AUC equal to 0.91. At the threshold of 47.7 Å^3^, it correctly predicted 86% of true positives with 14% of false positives. Treating false positives and false negatives as equally damaging, the distance based maximal clash with the protein was detected as the best predictor. The classification achieves best results for a threshold of 1.1 Å (the rates of true and false positives are 90 and 9%). The predictors of methylation mediated protection did not contain adjustable parameters (other than the clash threshold). Moreover, only a few clash measures were tested and almost all proved equally predictive. Therefore, we expect similar true and false positive rates for newly determined structures as for the already available ones. Each of the two measures, when applied to the protective non-cognate methyl groups correctly predicted 69% of cases. The number grows to 75% if both measures are applied and at least one of them gives a positive result. We think that the discrepancy of the results for cognate and non-cognate protective methylation might reflect more stringent protection by biologically relevant methylation compared to methylation that is only ‘incidentally’ protective.

### Protective methylation with little steric conflict

Steric conflicts predict the effect of methylation on susceptibility of DNA to cleavage, but not without exceptions. Grafting of protective methyl groups caused very limited steric conflict in the PvuII and Ecl18kI DNA complex structures, with the most drastic clashes of only 0.4 Å and 0.7 Å, respectively (compared to an average of 1.7 Å and a median of 1.8 Å for all other cases of protective methylation). PvuII and Ecl18kI also had the lowest sums of clashes and numbers of contacts, so the lack of one serious clash is in neither of the two cases compensated by a crowded environment of the protective methyl group.

In the case of PvuII, the only contact of the protective cytosine N4 methyl group (CAG^∧^m4CTG) is to His84 residue. The authors of the first structure of this enzyme hypothesize that the methyl would move His84 away, disrupt its hydrogen bond with guanine within the recognition sequence and in this way prevent cleavage (20). There is a structure of PvuII in complex with 5-iodocytosine containing DNA that was supposed to mimic cognate methylation to some degree. Unfortunately, the electron density in this particular protein region is unclear and the key residues are present in multiple conformations (21). The N4-methylated DNA is still bound and cleaved by PvuII (albeit with very low efficiency), and thus in case of this enzyme the discrimination between unmodified and methylated DNA might not be perfect (22).

The cognate methylation of Ecl18kI was not as extensively studied. The cytosine C5 methyl group (^∧^Cm5CNGG) clashes only with the Gln187 and Arg188 residues (23). Both contribute to the DNA binding and form three hydrogen bonds, one with the methylated cytosine and two with the neighboring guanine (24). We expect that in this case the energetic penalty of hydrogen bonds disruption is more pronounced and might be sufficient to account for the protective effect of the methyl group.

### Non-protective methylation with serious steric conflict

On the other end of the spectrum we have three cases of methyl groups with very serious clashes, although methylation in these positions was experimentally proven to be tolerated and not to affect cleavage. These are two N6 methyl groups of adenines in the PacI recognition sequence (most drastic clash of 2.2 and 1.7 Å) and one C5 methyl group of cytosine in BglI complex (1.5 Å). In comparison, the average most drastic clash for all other tolerated mutations equals 0.6 Å and has a median of 0.7 Å.

In the case of PacI enzyme, separate methylations of the first, second and last adenine in its TTAAT^∧^TAA target sequence (A3, A4 and A8) do not prevent DNA cleavage. The methyl group on A4 does not clash with protein, but the A3 and A8 methyl groups cause a steric conflict when modeled in *trans*. A3 forms hydrogen bonds via its Hoogsteen edge rather than the Watson–Crick edge. Therefore, its N6 methyl group should almost certainly be modeled in the *cis* form (toward the Watson–Crick edge), to avoid interference with base pairing.

A8 is not base paired, and thus also in this case the N6 methyl group can adopt the *cis* conformation. Modeling the A3 and A8 methyl groups in *cis* relieves the reported clashes, without introducing new significant ones (Supplementary Figure S6).

The C5 cytosine methyl group of non-protective BglI methylation (GCm5CNNNN^∧^NGGC) clashes only with the Asp268 residue, which is not involved in DNA binding or catalysis and situated on the periphery of the protein. It seems plausible that a conformational change of this residue would not substantially affect the structure and function of the enzyme.

## CONCLUSIONS

The statistical analysis presented in this work demonstrates that methyl groups protecting against restriction endonuclease cleavage have more steric conflict with the enzymes than those that do not block the reaction. Moreover, we have shown that the clash scores derived from structural data alone can be used to predict whether a methyl group would inhibit a given restriction endonuclease or not.

Our analysis is based on a few simplifications. We treated susceptibility versus resistance to DNA cleavage as a binary alterative (which might be the reason for the PvuII misprediction). We placed N6 and N4 methyl groups on the Hoogsteen edge without regard to the type of DNA base pairing (explaining the PacI misprediction). We treated hydrogen atoms implicitly and did not attempt to model the adaptive fit (perhaps accounting for the BglI misprediction). Finally, we ignored the loss of favorable hydrogen bonding interactions due to DNA methylation (most likely explaining the Ecl18kI misprediction). Nonetheless, the predictive success of clash analysis highlights the importance of steric conflict for the modulation of DNA cleavage by methylation.

Restriction endonucleases provide an excellent testbed for analyzing the effects of DNA methylation, because many accurate crystal structures are available and the REBASE database contains extensive ‘black and white’ data on how methylation affects endonuclease activity. It is likely that DNA cleavage efficiencies reflect at least in part DNA binding, which is less well documented. As we have not systematically tested this assumption, we recommend caution when applying clash scores from this manuscript to judge the effect of methylation on the interactions between proteins and ‘naked’ DNA (free from nucleosomes). Assessment of the role of methylation in a chromatin context in eukaryotes is still more difficult, because direct effects (as analyzed in this work) are likely to be confounded by indirect effects of chromatin structure. ENCODE CHIP-Seq and bisulfite data (25) contain comprehensive information about methylation dependent DNA binding of eukaryotic proteins (e.g. transcription factors), and many relevant crystal structures are available from the PDB. Thus, there may be opportunity for a similar analysis that could test whether clash scores may also be useful to predict *in vivo* effects in a chromatin context.

## SUPPLEMENTARY DATA

Supplementary Data are available at NAR Online.

SUPPLEMENTARY DATA
