# Supplementary material for: On the role of steric clashes in methylation control of restriction endonuclease activity
Source: Nucleic Acids Res. 2015 Dec 3;44(1):485–95. doi: 10.1093/nar/gkv1341 (PMC4705667; doi:10.1093/nar/gkv1341)
Supplement: SUPPLEMENTARY DATA [file supp_44_1_485__index.html]

On the role of steric clashes in methylation control of restriction endonuclease activity — On the role of steric clashes in methylation control of restriction endonuclease activity — SUPPLEMENTARY DATA 

# On the role of steric clashes in methylation control of restriction endonuclease activity

## SUPPLEMENTARY DATA

- SUPPLEMENTARY DATA
