# Supplementary material for: On the role of steric clashes in methylation control of restriction endonuclease activity
Source: Nucleic Acids Res. 2015 Dec 3;44(1):485–95. doi: 10.1093/nar/gkv1341 (PMC4705667; doi:10.1093/nar/gkv1341)
Supplement: SUPPLEMENTARY DATA [file supp_gkv1341_nar-03079-h-2015-File010.pdf]

Supplementary material for

On the role of steric clashes in methylation control of restriction endonuclease activity

Karolina Mierzejewska[1], Matthias Bochtler[1,2,#], Honorata Czapinska[1,2,#]

[1]*International Institute of Molecular and Cell Biology, Trojdena 4, 02-109 Warsaw, Poland*

[2]*Institute of Biochemistry and Biophysics PAS, Pawinskiego 5a, 02-106 Warsaw, Poland*

#Corresponding authors

phone: +48 22 592 5790, ++48 22 5970732

mail: honorata@iimcb.gov.pl, mbochtler@iimcb.gov.pl

Running title: Significance of steric clashes in RM systems

Supplementary figures

Figure S1

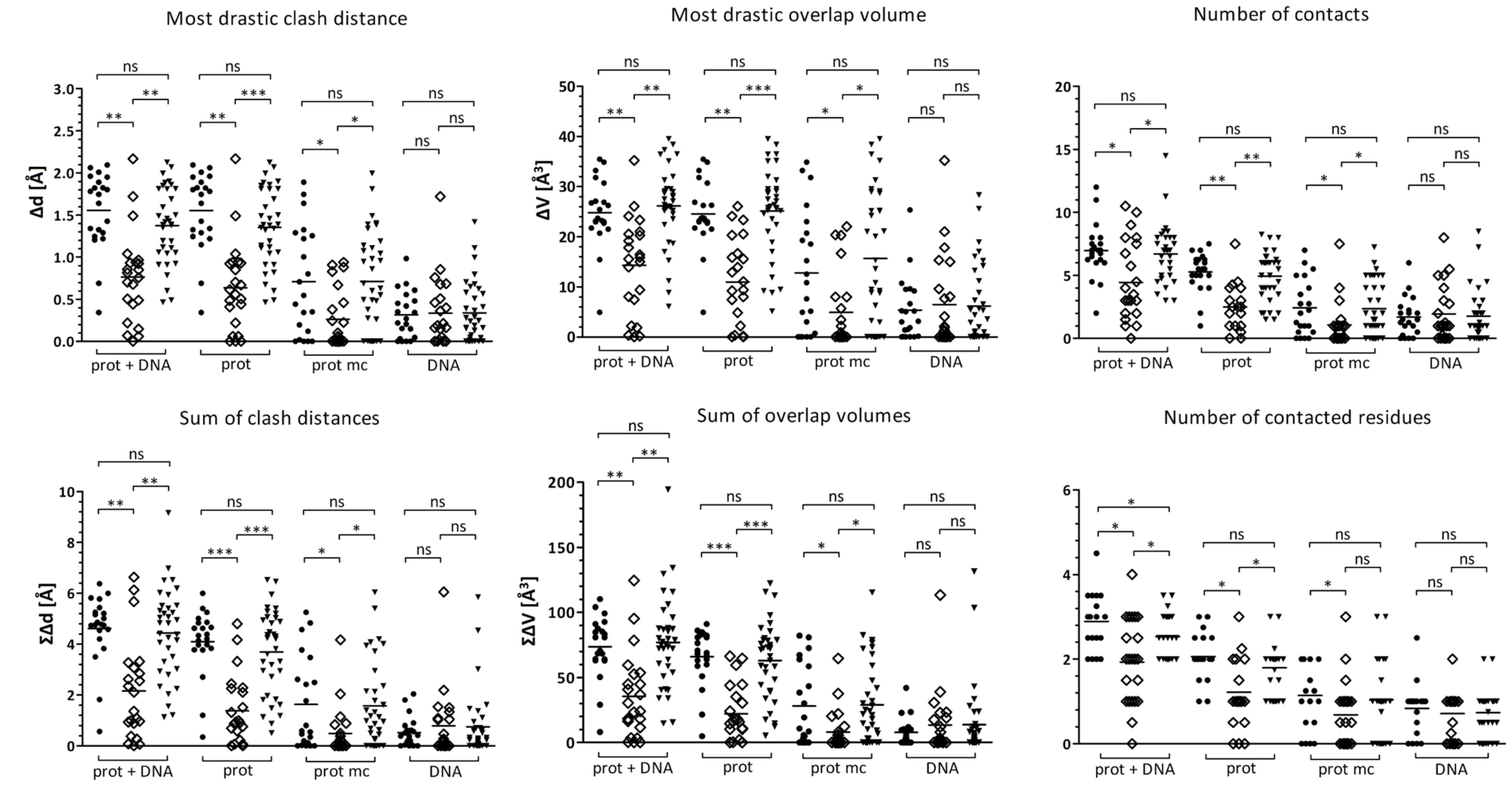

● cognate methylation
◊ tolerated methylation
▼ protective non-cognate methylation

Wilcoxon rank sum test p-values:
ns – not significant
* p≤0.05
** p≤0.0005
*** p≤0.000005

Figure S1. Statistical analysis of steric clashes with the *in silico* introduced methyl groups. Each dot represents one enzyme for which a representative structure was chosen and methyl groups from both strands as well as from multiple molecules in asymmetric unit were averaged. Prot - protein, prot mc - protein main chain.

Figure S2

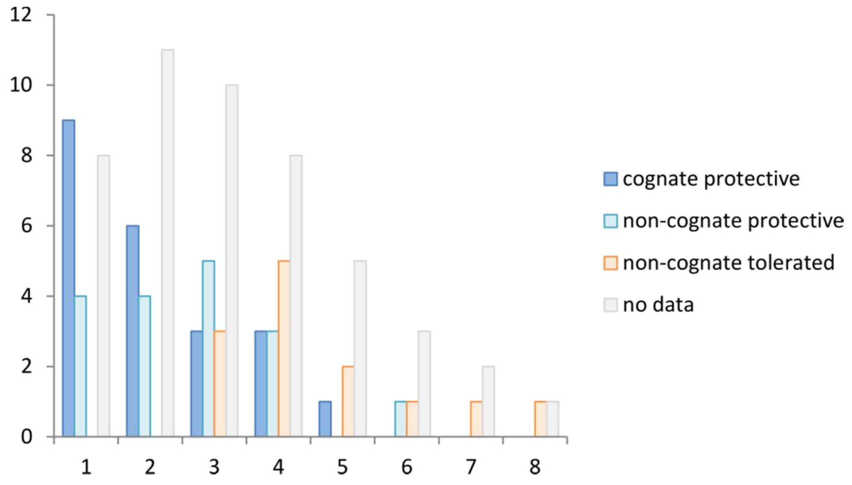

Figure S2. Effect of the most clashing methyl groups on the enzyme activity. For each enzyme all possible methylation types in the recognized sequence were modelled and the methyl groups were ordered according to the clash magnitude (from most to least clashing ones). Next, the occurrence of protective (cognate and non-cognate) and tolerated methylations was assessed.

Figure S3

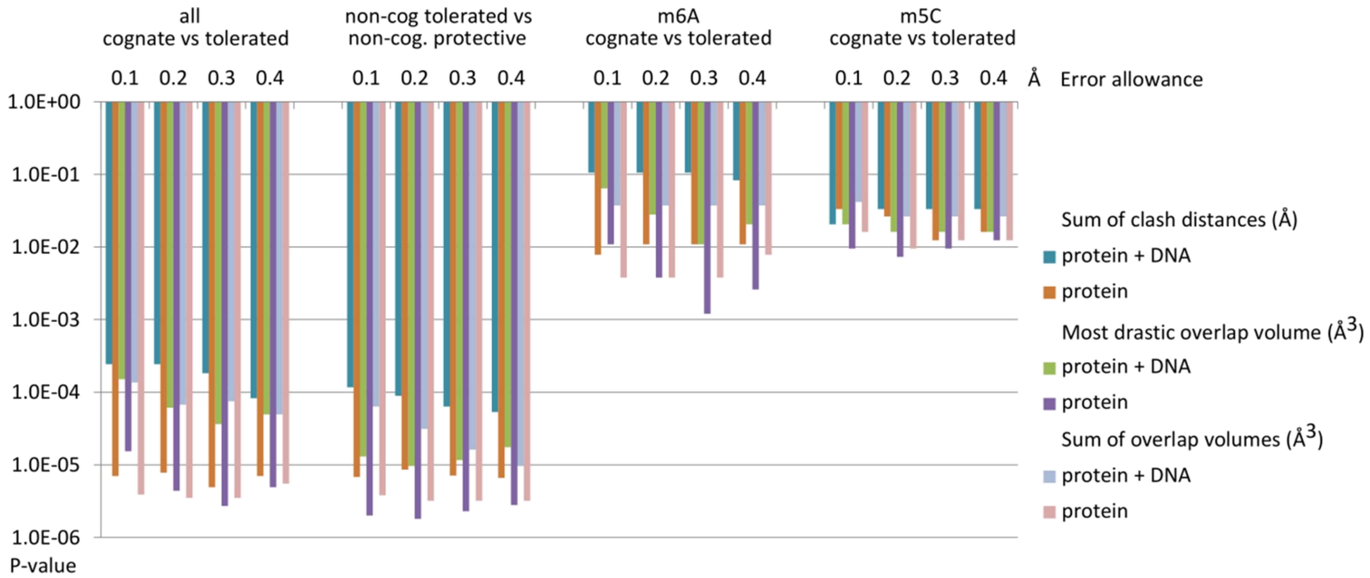

Figure S3. The effect of coordinate error and adaptive fit on the properties of analyzed clash measures. The increasing error allowance was deduced from the interatomic distances prior to clash estimation. The Wilcoxon rank sum test was used to estimate the p-values for the selected clash measures. The most drastic clash distance was not affected and thus not included in the figure (see also Table S4).

Figure S4

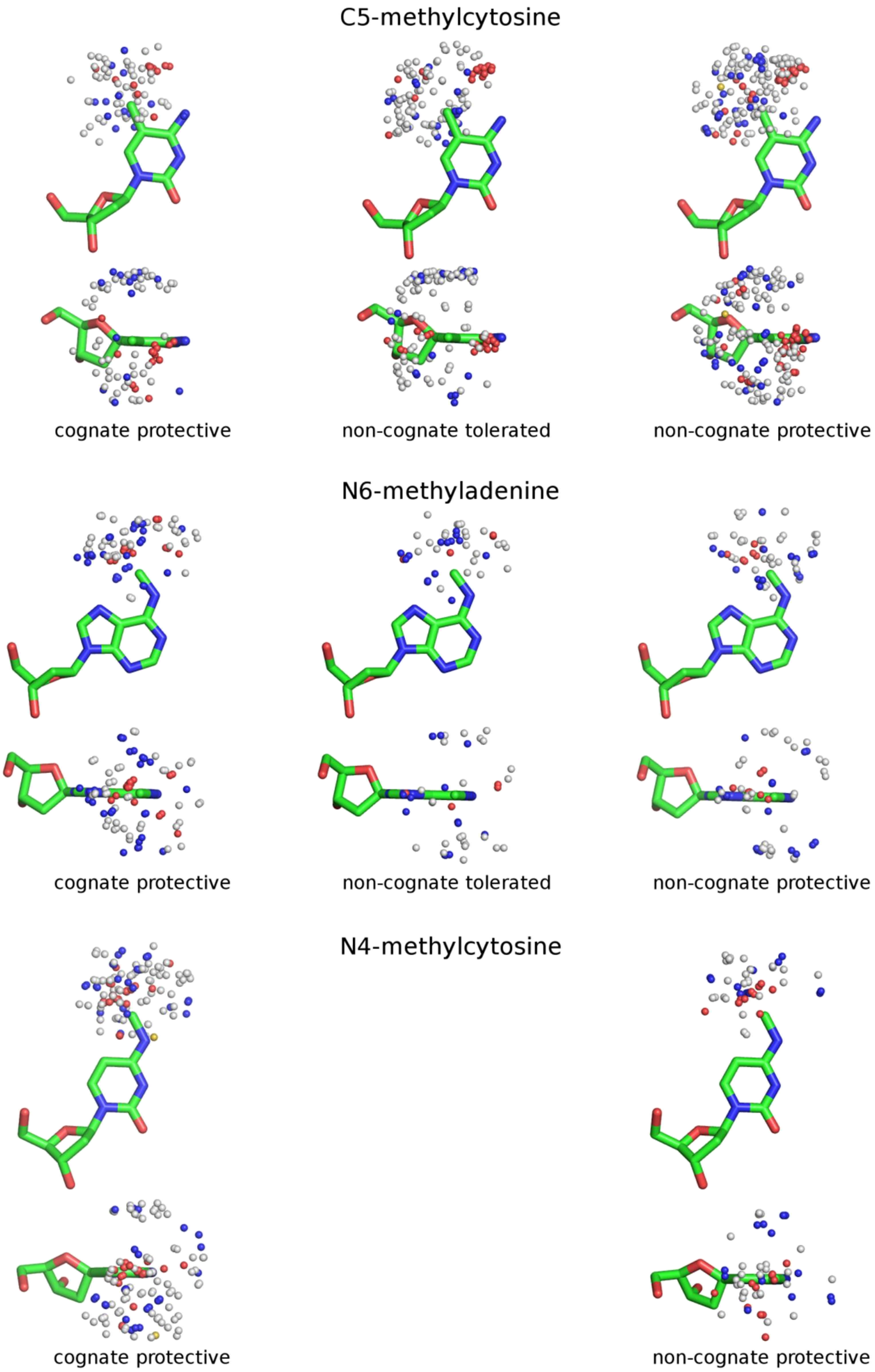

Figure S4. Atoms closer than the sum of the van der Waals radii to methyl groups *in silico* introduced in the restriction endonuclease-DNA complex structures. The methyl groups were modelled in positions corresponding to cognate protective methylation and methylation experimentally proven to be tolerated by the enzyme. The clashing atoms are colored according to the atom type. Only one molecule in the asymmetric unit was chosen. The clashes of the methyl groups on both DNA strands are included.

Figure S5

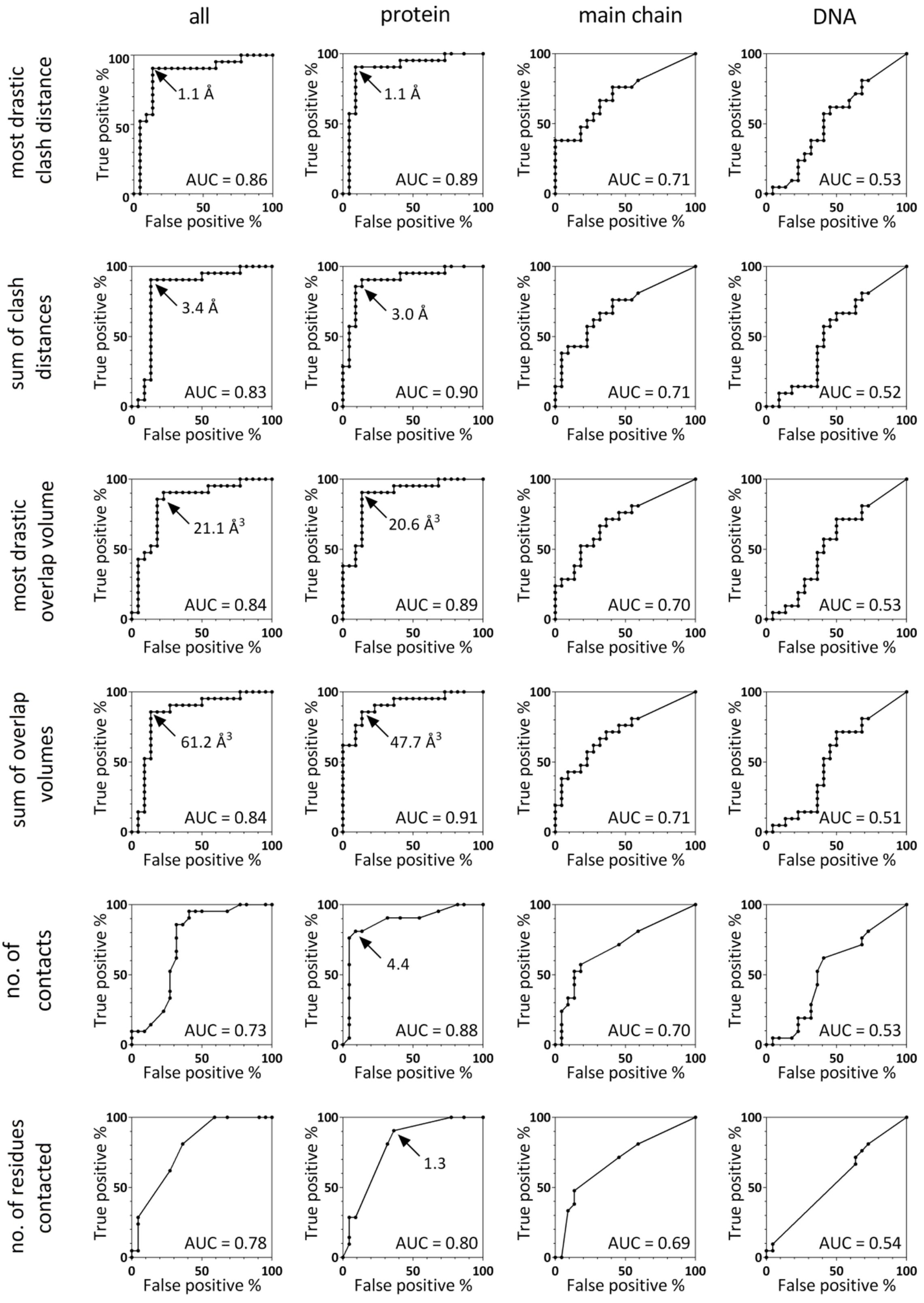

Figure S5. Receiver Operating Characteristic (ROC) curves. The ROC curves were calculated using cognate protective and non-cognate tolerated cases. AUC – areas under the curves. For the best predictor AUC is close to 1, AUC close to 0.5 means random predictor. For cases with AUC > 0.8 thresholds with approximately equal true positive to false positive ratios are indicated.

Figure S6

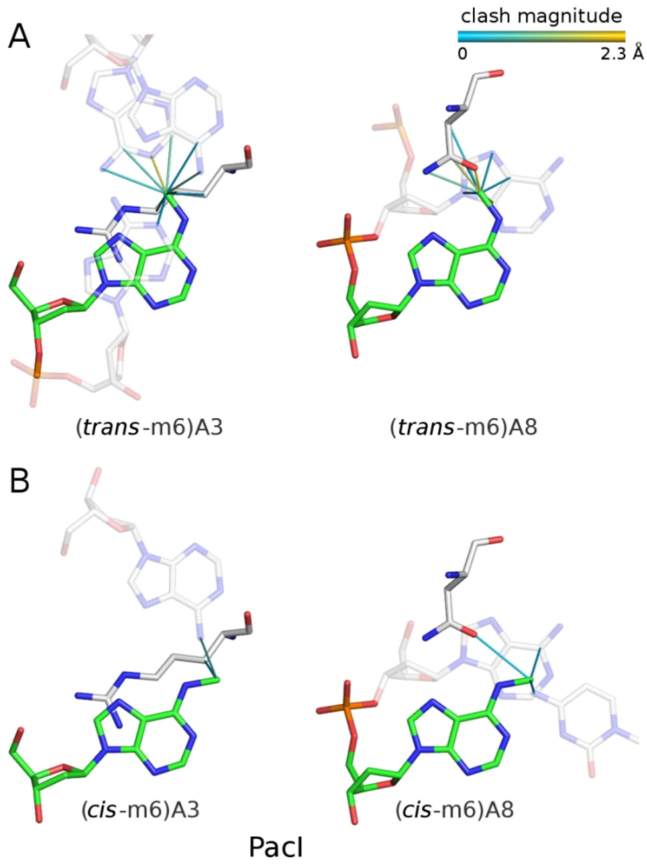

Figure S6. Methyl group clashes in the structure of PacI-DNA complex (1). The methyl groups tolerated on adenines in the TTAAT^TAA PacI target sequence (A3 and A8) have been modelled in (A) *trans* and (B) *cis* configuration. Due to the DNA distortion (absence of canonical Watson-Crick pairing), the methyl groups of the two modified adenines most likely adopt the *cis* conformation, which results in almost no steric clashes in contrast to the situation when they would be present in the *trans* position.

1. Shen, B.W., Heiter, D.F., Chan, S.H., Wang, H., Xu, S.Y., Morgan, R.D., Wilson, G.G. and Stoddard, B.L. (2010) Unusual target site disruption by the rare-cutting HNH restriction endonuclease PacI. *Structure*, **18**, 734.

Supplementary tables

Table S1. Restriction endonucleases used in the study ordered according to the modification introduced by their cognate methyltransferases. The target sequences and methylation sensitivity were taken from REBASE database (http://rebase.neb.com).

REase name	PDB code	Res. [Å]	REase target sequence	Cognate methylation	Non-cognate tolerated methylation	Non-cognate protective methylation
**C5-methylcytosine**						
Bse634I	3V1Z	2.20	R^CCGGY	m5C unknown		R^Cm5CGGY
Ecl18kI	2FQZ	2.0	^CCNGG	^Cm5CNGG		
EcoO109I	1WTE	1.90	RG^GNCCY	RG^GNm5CCY	RG^GNCCm5C<br>m6AG^GNCCY	RG^GNCm5CY
HinP1I	2FKC	2.39	G^CGC	G^m5CGC		G^CGm5C
MspI	1SA3	1.95	C^CGG	m5C^CGG	C^m5CGG	m4C^CGG
NaeI	1IAW	2.40	GCC^GGC	m5C unknown		Gm5CC^GGC<br>GCm5C^GGC<br>GCC^GGm5C
NgoMIV	4ABT	2.22	G^CCGGC	G^m5CCGGC		G^Cm5CGGC<br>G^CCGGm5C
SgrAI	3DVO	1.89	CR^CCGGYG	m5C unknown		Cm6A^CCGGYG<br>CR^m5CCGGYG<br>CR^Cm5CGGYG
**N6-methyladenine**						
EcoRI	1CKQ	1.87	G^AATTC	G^Am6ATTC		G^m6AATTC<br>G^AATTm5C
EcoRV	1B94	1.90	GAT^ATC	Gm6AT^ATC	GAT^ATm5C	GAT^m6ATC
FokI	1FOK	2.80	GGATG (9/13)	5' GGm6ATG 3'<br>3' CCTm6AC 5'	5' GGATG 3'<br>3' Cm5CTAC 5'	
HincII	1TX3	2.50	GTY^RAC	GTY^Rm6AC	GTm5C^GAC	GTY^RAm5C
HindIII	2E52	2.0	A^AGCTT	m6A^AGCTT	A^m6AGCTT	A^AGm5CTT<br>A^AGm4CTT
Hpy188I	3OQG	1.75	TCN^GA	TCN^Gm6A		
MunI	1D02	1.70	C^AATTG	C^Am6ATTG		
**N4-methylcytosine**						
BamHI	2BAM	2.00	G^GATCC	G^GATm4CC	G^Gm6ATCC<br>G^GATCm5C	G^GATm5CC
BcnI	2ODI	1.45	CC^SGG	Cm4C^SGG	m5CC^SGG	CC^m4CGG
BfiI	3ZI5	3.20	ACTGGG (5/4)	5’ Am4CTGGG 3’<br>3’ TGACm4CC 5’		
BglI	1DMU	2.20	GCCN_4^NGGC	m4C unknown	GCm5CN_4^NGGC	Gm4CCN_4^NGGC<br>GCm4CN_4^NGGC<br>GCCN_4^NGGm5C
BglII	1DFM	1.50	A^GATCT	A^GATm4CT	A^Gm6ATCT	m6A^GATCT<br>A^GATm5CT
BsoBI	1DC1	1.70	C^YCGRG	m4C unknown	C^Ym5CGRG	C^Ym4CGRG<br>m4C^YCGRG
BstYI	1VRR	2.7	R^GATCY	R^GATm4CY	R^Gm6ATCY	
Hpy99I	3GOX	1.50	CGWCG^	CGWm4CG^		
MvaI	2OAA	1.5	CC^WGG	Cm4C^WGG	Cm5C^WGG	m4CC^WGG
NotI	3C25	2.50	GC^GGCCGC	m4C unknown	GC^GGCCGm5C	GC^GGm5CCGC
PspGI	3BM3	1.70	^CCWGG	m4C unknown		^Cm5CWGG
PvuII	1PVI	2.60	CAG^CTG	CAG^m4CTG		Cm6AG^CTG
SfiI	2EZV	2.40	GGCCN_4^NGGCC	m4C unknown	GGm5CCN_4^NGGCC<br>GGCCN_4^NGGm5CC<br>GGCCN_4^NGGCm5C	GGCm4CN_4^NGGCC<br>GGCm5CN_4^NGGCC
ThaI	3NDH	1.30	CG^CG	m4CG^CG		m5CG^CG
**No cognate methyltransferase**						
PacI	3LDY	1.97	TTAAT^TAA	no cognate MTase	TTm6AAT^TAA<br>TTAm6AT^TAA<br>TTAAT^TAm6A	TTAAT^Tm6AA

Table S2. Steric clashes of the *in silico* introduced methyl groups with the protein and DNA atoms in the restriction endonuclease-DNA complex structures. The values for methyl groups in both DNA strands, as well as for all copies in the crystallographic asymmetric units were averaged. Prot – protein; mc – main chain.

A. Modifications corresponding to the protection by cognate methyltransferase

			Most drastic clash distance (Å)				Sum of clash distances (Å)				Most drastic overlap volume (Å^3)				Sum of overlap volumes (Å^3)				Number of contacts				No of contacted residues			
enzyme	PDB ID	methylated sequence	prot+ DNA	prot	prot mc	DNA	prot+ DNA	prot	prot mc	DNA	prot+ DNA	prot	prot mc	DNA	prot+ DNA	prot	prot mc	DNA	prot+ DNA	prot	prot mc	DNA	prot+ DNA	prot	prot mc	DNA
**C5-methylcytosine**																										
Ecl18kI	2FQZ	^Cm5CNGG	0.69	0.69	0.00	0.35	1.82	1.21	0.00	0.62	15.4	15.4	0.0	5.1	29.1	21.5	0.0	7.7	4.3	2.0	0.0	2.3	3.0	2.0	0.0	1.0
EcoO109I	1WTE	RG^GNm5CCY	1.33	1.33	1.33	0.56	5.77	3.97	3.48	1.80	34.8	34.8	34.8	9.2	99.2	76.4	67.2	22.8	12.0	6.0	5.0	6.0	2.5	1.5	1.5	1.0
HinP1I	2FKC	G^m5CGC	1.21	1.15	0.71	0.45	4.54	3.97	1.77	0.57	25.4	23.7	15.2	9.5	80.9	70.6	30.1	10.3	7.5	5.5	3.5	2.0	3.0	2.0	2.0	1.0
MspI	1SA3	m5C^CGG	1.43	1.43	0.20	0.35	3.50	2.71	0.20	0.79	21.7	21.7	3.0	5.2	50.4	40.5	3.0	9.9	7.3	4.0	0.5	3.3	2.5	1.5	0.5	1.0
NgoMIV	4ABT	G^m5CCGGC	1.25	1.25	0.80	0.99	5.82	3.78	0.80	2.04	26.7	23.8	19.2	25.3	110.3	68.3	19.2	42.0	8.0	4.0	1.0	4.0	2.5	1.0	1.0	1.5
		**Average:**	**1.18**	**1.17**	**0.61**	**0.54**	**4.29**	**3.13**	**1.25**	**1.16**	**24.8**	**23.9**	**14.5**	**10.9**	**74.0**	**55.4**	**23.9**	**18.5**	**7.8**	**4.3**	**2.0**	**3.5**	**2.7**	**1.6**	**1.0**	**1.1**
**N6-methyladenine**																										
EcoRI	1CKQ	G^Am6ATTC	1.29	1.29	0.00	0.42	4.76	4.34	0.00	0.42	30.7	30.7	0.0	5.2	87.7	82.5	0.0	5.2	6.0	5.0	0.0	1.0	3.0	2.0	0.0	1.0
EcoRV	1B94	Gm6AT^ATC	1.95	1.95	0.02	0.00	4.21	4.21	0.02	0.00	23.2	23.2	0.0	0.0	63.1	63.1	0.0	0.0	4.5	4.5	0.5	0.0	2.0	2.0	0.5	0.0
FokI	1FOK	5' GGm6ATG 3'<br>3' CCTm6AC 5'	2.10	2.10	0.00	0.27	5.01	4.62	0.00	0.39	23.7	23.7	0.0	3.1	64.0	59.9	0.0	4.0	8.0	6.5	0.0	1.5	3.5	2.5	0.0	1.0
HincII	1TX3	GTY^Rm6AC	1.80	1.80	0.44	0.03	5.44	5.40	0.58	0.03	26.8	26.8	7.6	0.2	83.3	83.1	8.6	0.2	6.5	6.3	2.3	0.3	3.3	3.0	2.0	0.3
HindIII	2E52	m6A^AGCTT	1.96	1.96	0.35	0.61	4.74	4.13	0.46	0.61	23.1	23.1	5.1	9.6	68.4	58.8	5.8	9.6	6.3	5.0	2.0	1.3	3.0	2.0	2.0	1.0
Hpy188I	3OQG	TCN^Gm6A	1.83	1.83	1.26	0.00	4.86	4.86	2.42	0.00	33.2	33.2	33.2	0.0	91.1	91.1	58.6	0.0	6.0	6.0	4.0	0.0	2.0	2.0	1.0	0.0
MunI	1D02	C^Am6ATTG	1.34	1.34	0.00	0.48	5.16	4.66	0.00	0.51	35.4	35.4	0.0	6.6	92.7	86.0	0.0	6.6	9.0	7.0	0.0	2.0	3.0	2.0	0.0	1.0
		**Average:**	**1.75**	**1.75**	**0.30**	**0.26**	**4.88**	**4.60**	**0.50**	**0.28**	**28.0**	**28.0**	**6.6**	**3.5**	**78.6**	**74.9**	**10.4**	**3.7**	**6.6**	**5.8**	**1.3**	**0.9**	**2.8**	**2.2**	**0.8**	**0.6**
**N4-methylcytosine**																										
BamHI	2BAM	G^GATm4CC	1.75	1.75	1.75	0.69	6.37	5.01	4.58	1.37	27.1	26.3	26.3	15.4	104.1	80.7	72.6	23.4	11.0	7.5	6.0	3.5	3.5	2.5	2.0	1.0
BcnI	2ODI	Cm4C^SGG	1.82	1.82	1.29	0.15	4.58	4.38	2.61	0.20	21.5	21.5	18.5	1.4	68.4	66.8	43.3	1.6	6.8	5.5	2.8	1.3	3.5	2.8	1.3	0.8
BfiI	3ZI5	5' Am4CTGGG 3'<br>3' TGACm4CC 5'	2.06	2.06	0.55	0.00	6.00	6.00	0.55	0.00	26.2	26.2	10.8	0.0	84.8	84.8	10.8	0.0	6.3	6.3	1.3	0.0	2.5	2.5	1.0	0.0
BglII	1DFM	A^GATm4CT	1.89	1.89	1.89	0.00	5.25	5.25	5.25	0.00	23.0	23.0	23.0	0.0	80.9	80.9	80.9	0.0	7.0	7.0	7.0	0.0	2.0	2.0	2.0	0.0
BstYI	1VRR	R^GATm4CY	1.22	1.22	1.22	0.05	3.83	3.77	3.77	0.06	20.7	20.7	20.7	0.1	64.1	64.0	64.0	0.1	7.0	5.5	5.5	1.5	3.0	2.0	2.0	1.0
Hpy99I	3GOX	CGWm4CG^	2.01	2.01	0.12	0.66	4.73	3.83	0.12	0.90	22.7	22.7	0.7	10.7	62.9	50.9	0.7	12.0	7.5	5.0	1.0	2.5	4.5	2.0	1.0	2.5
MvaI	2OAA	Cm4C^WGG	1.78	1.78	1.01	0.15	4.77	4.62	2.49	0.15	22.9	22.9	12.5	1.5	70.5	69.0	38.3	1.5	6.8	6.3	2.8	0.5	3.5	3.0	1.3	0.5
PvuII	1PVI	CAG^m4CTG	0.35	0.35	0.35	0.22	0.57	0.35	0.35	0.22	5.0	5.0	5.0	3.0	8.0	5.0	5.0	3.0	2.0	1.0	1.0	1.0	2.0	1.0	1.0	1.0
ThaI	3NDH	m4CG^CG	1.64	1.64	1.64	0.21	5.21	4.83	4.83	0.38	31.8	31.8	31.8	1.9	85.3	82.2	82.2	3.1	7.0	5.0	5.0	2.0	3.0	2.0	2.0	1.0
		**Average:**	**1.61**	**1.61**	**1.09**	**0.23**	**4.59**	**4.23**	**2.73**	**0.36**	**22.3**	**22.2**	**16.6**	**3.8**	**69.9**	**64.9**	**44.2**	**5.0**	**6.8**	**5.4**	**3.6**	**1.4**	**3.1**	**2.2**	**1.5**	**0.9**
		**Average of all 3 modifications:**	**1.56**	**1.55**	**0.71**	**0.31**	**4.61**	**4.09**	**1.63**	**0.53**	**24.8**	**24.6**	**12.7**	**5.4**	**73.8**	**66.0**	**28.1**	**7.8**	**7.0**	**5.3**	**2.4**	**1.7**	**2.9**	**2.1**	**1.1**	**0.8**

B. Modifications experimentally proven to be tolerated

			Most drastic clash distance (Å)				Sum of clash distances (Å)				Most drastic overlap volume ($Å^3$)				Sum of overlap volumes ($Å^3$)				Number of contacts				No of contacted residues			
enzyme	PDB ID	methylated sequence	prot+ DNA	prot	prot mc	DNA	prot+ DNA	prot	prot mc	DNA	prot+ DNA	prot	prot mc	DNA	prot+ DNA	prot	prot mc	DNA	prot+ DNA	prot	prot mc	DNA	prot+ DNA	prot	prot mc	DNA
**C5-methylcytosine**																										
BamHI	2BAM	G^GATCm5C	1.04	1.04	0.10	0.51	2.85	1.32	0.10	1.54	15.6	15.6	0.9	9.6	40.8	17.8	0.9	23.0	7.5	2.5	0.5	5.0	2.0	1.0	0.5	1.0
BcnI	2ODI	m5CC^SGG	0.93	0.93	0.91	0.17	2.62	2.44	2.04	0.18	20.5	20.5	20.4	1.1	45.0	43.8	37.4	1.1	5.8	4.5	4.0	1.3	3.0	2.0	2.0	1.0
BglI	1DMU	GCm5CN$_4$^NGGC	1.49	1.49	0.83	0.00	3.32	3.32	0.83	0.00	20.3	20.3	20.3	0.0	59.6	59.6	20.3	0.0	3.0	3.0	1.0	0.0	1.0	1.0	1.0	0.0
BsoBI	1DC1	C^Ym5CGRG	0.49	0.41	0.00	0.46	1.36	0.90	0.00	0.46	8.0	4.8	0.0	8.0	18.3	10.3	0.0	8.0	3.5	2.5	0.0	1.0	3.0	2.0	0.0	1.0
EcoO109I	1WTE	RG^GNCCm5C	0.22	0.22	0.00	0.16	0.38	0.22	0.00	0.16	2.2	2.2	0.0	1.2	3.5	2.2	0.0	1.2	2.0	1.0	0.0	1.0	2.0	1.0	0.0	1.0
EcoRV	1B94	GAT^ATm5C	0.71	0.71	0.05	0.22	0.97	0.76	0.05	0.22	15.8	15.8	0.1	2.1	18.1	15.9	0.1	2.1	3.0	2.0	1.0	1.0	3.0	2.0	1.0	1.0
FokI	1FOK	5' GGATG 3'	0.76	0.67	0.67	0.76	2.55	1.51	1.14	1.04	17.8	8.0	8.0	17.8	36.9	17.9	12.3	19.0	8.0	4.0	3.0	4.0	3.0	2.0	1.0	1.0
HincII	1TX3	GTm5C^GAC	0.92	0.92	0.88	0.05	2.34	2.29	0.88	0.05	23.3	23.3	22.0	0.5	45.0	44.5	22.0	0.5	4.5	4.3	1.0	0.3	2.5	2.3	1.0	0.3
MspI	1SA3	C^m5CGG	0.82	0.49	0.23	0.40	3.28	2.25	0.23	1.03	16.4	9.2	2.2	7.7	52.6	35.2	2.2	17.4	6.8	4.0	0.5	2.8	1.5	0.5	0.5	1.0
MvaI	2OAA	Cm5C^WGG	0.68	0.62	0.46	0.68	3.07	0.88	0.48	2.19	15.0	12.9	8.0	15.0	53.6	14.5	8.0	39.1	8.0	2.5	1.5	5.5	2.5	1.5	1.5	1.0
NotI	3C25	GC^GGCCGm5C	0.86	0.00	0.00	0.86	1.09	0.00	0.00	1.09	21.0	0.0	0.0	21.0	23.3	0.0	0.0	23.3	2.0	0.0	0.0	2.0	1.0	0.0	0.0	1.0
SfiI	2EZV	GGCCN$_4$^NGGm5CC	0.06	0.06	0.00	0.00	0.06	0.06	0.00	0.00	0.2	0.2	0.0	0.0	0.2	0.2	0.0	0.0	1.0	1.0	0.0	0.0	1.0	1.0	0.0	0.0
SfiI	2EZV	GGCCN$_4$^NGGCm5C	0.15	0.00	0.00	0.15	0.27	0.00	0.00	0.27	1.9	0.0	0.0	1.9	3.2	0.0	0.0	3.2	1.0	0.0	0.0	1.0	0.5	0.0	0.0	0.5
SfiI	2EZV	GGm5CCN$_4$^NGGCC	0.97	0.94	0.94	0.69	5.68	4.17	4.17	1.51	19.2	16.7	16.7	15.3	95.3	64.6	64.6	30.7	10.5	7.5	7.5	3.0	4.0	3.0	3.0	1.0
		**Average:**	**0.72**	**0.61**	**0.36**	**0.36**	**2.13**	**1.44**	**0.71**	**0.69**	**14.1**	**10.7**	**7.0**	**7.2**	**35.4**	**23.3**	**12.0**	**12.0**	**4.8**	**2.8**	**1.4**	**2.0**	**2.1**	**1.4**	**0.8**	**0.8**
**N6-methyladenine**																										
BamHI	2BAM	G^Gm6ATCC	0.07	0.06	0.06	0.02	0.08	0.06	0.06	0.02	0.3	0.3	0.3	0.0	0.3	0.3	0.3	0.0	1.5	0.5	0.5	1.0	1.5	0.5	0.5	1.0
BglII	1DFM	A^Gm6ATCT	0.85	0.85	0.00	0.00	2.11	2.11	0.00	0.00	13.9	13.9	0.0	0.0	30.5	30.5	0.0	0.0	4.0	4.0	0.0	0.0	2.0	2.0	0.0	0.0
BstYI	1VRR	R^Gm6ATCY	0.51	0.51	0.25	0.00	0.99	0.99	0.25	0.00	9.4	9.4	3.4	0.0	16.3	16.3	3.4	0.0	3.0	3.0	1.0	0.0	1.0	1.0	1.0	0.0
EcoO109I	1WTE	m6AG^GNCCY	0.94	0.94	0.00	0.00	0.94	0.94	0.00	0.00	24.1	24.1	0.0	0.0	24.1	24.1	0.0	0.0	1.0	1.0	0.0	0.0	1.0	1.0	0.0	0.0
HindIII	2E52	A^m6AGCTT	0.44	0.44	0.00	0.19	0.88	0.69	0.00	0.19	7.5	7.5	0.0	1.3	11.6	10.3	0.0	1.3	3.0	2.0	0.0	1.0	2.0	1.0	0.0	1.0
PacI	3LDY	TTAAT^TAm6A	2.17	2.17	0.38	0.36	6.13	4.80	0.38	1.33	26.1	26.1	5.7	4.1	78.5	66.4	5.7	12.1	9.0	4.0	1.0	5.0	2.0	1.0	1.0	1.0
PacI	3LDY	TTm6AAT^TAA	1.72	0.56	0.02	1.72	6.63	0.58	0.02	6.05	35.2	11.0	0.0	35.2	124.5	11.1	0.0	113.5	10.0	2.0	1.0	8.0	3.0	1.0	1.0	2.0
PacI	3LDY	TTAm6AT^TAA	0.00	0.00	0.00	0.00	0.00	0.00	0.00	0.00	0.0	0.0	0.0	0.0	0.0	0.0	0.0	0.0	0.0	0.0	0.0	0.0	0.0	0.0	0.0	0.0
		**Average:**	**0.84**	**0.69**	**0.09**	**0.29**	**2.22**	**1.27**	**0.09**	**0.95**	**14.6**	**11.5**	**1.2**	**5.1**	**35.7**	**19.9**	**1.2**	**15.9**	**3.9**	**2.1**	**0.4**	**1.9**	**1.6**	**0.9**	**0.4**	**0.6**
		**Average of 2 modifications:**	**0.76**	**0.64**	**0.26**	**0.34**	**2.16**	**1.38**	**0.48**	**0.79**	**14.3**	**11.0**	**4.9**	**6.4**	**35.5**	**22.1**	**8.1**	**13.4**	**4.5**	**2.5**	**1.1**	**1.9**	**1.9**	**1.2**	**0.7**	**0.7**

C. Modifications experimentally proven to be protective but not covered by cognate methyltransferase

			Most drastic clash distance (Å)				Sum of clash distances (Å)				Most drastic overlap volume ($Å^3$)				Sum of overlap volumes ($Å^3$)				Number of contacts				No of contacted residues			
enzyme	PDB ID	methylated sequence	prot+ DNA	prot	prot mc	DNA	prot+ DNA	prot	prot mc	DNA	prot+ DNA	prot	prot mc	DNA	prot+ DNA	prot	prot mc	DNA	prot+ DNA	prot	prot mc	DNA	prot+ DNA	prot	prot mc	DNA
**C5-methylcytosine**																										
BamHI	2BAM	G^GATm5CC	1.05	1.05	1.05	0.41	5.51	4.88	4.20	0.63	25.2	25.2	25.2	6.5	106.1	97.8	82.5	8.3	8.0	6.0	5.0	2.0	3.0	2.0	1.0	1.0
BglI	1DMU	GCCN$_4$^NGGm5C	1.19	1.19	1.19	0.00	4.82	4.82	4.01	0.00	29.5	29.5	29.5	0.0	85.4	85.4	76.5	0.0	7.0	7.0	5.0	0.0	2.0	2.0	2.0	0.0
BglII	1DFM	A^GATm5CT	0.60	0.60	0.60	0.57	2.33	1.62	1.62	0.71	11.2	9.5	9.5	11.2	34.0	21.9	21.9	12.2	7.5	5.5	5.5	2.0	3.0	2.0	2.0	1.0
Bse634I	3V1Z	R^Cm5CGGY	0.79	0.68	0.00	0.68	2.25	0.88	0.00	1.37	15.3	9.2	0.0	15.2	40.5	12.4	0.0	28.1	4.5	1.5	0.0	3.0	2.5	1.5	0.0	1.0
EcoO109I	1WTE	RG^GNCm5CY	1.43	1.34	1.34	1.11	9.16	3.32	2.95	5.85	37.3	35.1	35.1	28.3	194.5	63.0	59.2	131.5	14.5	6.0	4.0	8.5	2.0	1.0	1.0	1.0
EcoRI	1CKQ	G^AATTm5C	0.91	0.91	0.69	0.00	3.31	3.31	2.13	0.00	18.9	18.9	15.3	0.0	53.8	53.8	31.8	0.0	7.0	7.0	5.0	0.0	2.0	2.0	1.0	0.0
HincII	1TX3	GTY^RAm5C	1.11	1.11	1.11	0.05	4.25	4.21	4.07	0.05	29.1	29.1	29.1	0.2	73.5	73.3	72.2	0.2	8.8	8.3	7.3	0.5	3.5	3.0	3.0	0.5
HindIII	2E52	A^AGm5CTT	0.91	0.91	0.00	0.16	2.94	2.72	0.00	0.22	17.0	17.0	0.0	0.9	40.8	39.7	0.0	1.1	8.3	6.3	0.0	2.0	3.3	2.3	0.0	1.0
HinP1I	2FKC	G^CGm5C	1.20	1.14	1.08	0.80	4.06	2.95	2.15	1.11	28.2	21.0	21.0	18.9	72.3	48.4	36.7	23.9	7.5	5.0	4.0	2.5	2.0	1.0	1.0	1.0
NaeI	1IAW	GCC^GGm5C	1.50	1.50	0.95	0.28	3.51	3.19	1.40	0.32	32.0	32.0	20.1	5.4	63.7	58.0	28.4	5.7	4.5	3.5	1.5	1.0	2.5	1.8	0.8	0.8
NaeI	1IAW	Gm5CC^GGC	1.60	1.60	0.96	0.19	6.20	5.95	0.96	0.25	27.1	27.1	24.2	2.9	116.5	113.1	24.2	3.5	7.3	6.5	1.0	0.8	2.0	1.5	1.0	0.5
NaeI	1IAW	GCm5C^GGC	1.06	0.80	0.00	1.00	6.51	1.97	0.00	4.54	26.1	14.4	0.0	25.6	134.2	30.7	0.0	103.5	11.3	4.0	0.0	7.3	3.0	2.0	0.0	1.0
NgoMIV	4ABT	G^Cm5CGGC	0.93	0.82	0.00	0.71	2.80	1.19	0.00	1.62	18.7	11.9	0.0	16.1	50.9	17.5	0.0	33.4	5.0	2.0	0.0	3.0	3.0	2.0	0.0	1.0
NgoMIV	4ABT	G^CCGGm5C	1.38	1.38	0.63	0.13	3.84	3.61	0.63	0.23	22.4	22.4	13.6	1.1	60.8	59.0	13.6	1.8	5.5	4.5	1.0	1.0	2.5	2.0	1.0	0.5
NotI	3C25	GC^GGm5CCGC	1.49	1.49	1.49	0.29	4.15	3.82	1.78	0.33	38.4	38.4	38.4	3.5	79.5	75.9	41.9	3.6	6.0	4.0	2.0	2.0	2.0	1.0	1.0	1.0
PspGI	3BM3	^Cm5CWGG	1.06	1.06	0.00	0.16	2.04	1.83	0.00	0.21	27.9	27.9	0.0	1.1	40.2	38.9	0.0	1.3	4.0	2.0	0.0	2.0	3.0	2.0	0.0	1.0
SfiI	2EZV	GGCm5CN4^NGGCC	1.39	1.39	0.98	0.00	4.38	4.38	0.98	0.00	36.5	36.5	25.1	0.0	87.9	87.9	25.1	0.0	6.5	6.5	1.0	0.0	3.0	3.0	1.0	0.0
SgrAI	3DVO	CR^m5CCGGYG	1.46	1.46	0.86	0.63	5.94	4.47	0.86	1.47	25.8	25.8	21.1	13.3	101.7	78.2	21.1	23.5	8.5	4.0	1.0	4.5	3.0	1.0	1.0	2.0
SgrAI	3DVO	CR^Cm5CGGYG	0.47	0.47	0.00	0.37	1.21	0.51	0.00	0.71	6.1	5.2	0.0	5.3	15.4	5.3	0.0	10.1	3.5	1.5	0.0	2.0	2.5	1.5	0.0	1.0
ThaI	3NDH	m5CG^CG	1.39	1.39	1.39	0.00	6.53	6.53	3.72	0.00	29.0	29.0	29.0	0.0	115.8	115.8	67.9	0.0	8.0	8.0	4.0	0.0	2.0	2.0	1.0	0.0
		**Average:**	**1.14**	**1.11**	**0.72**	**0.38**	**4.29**	**3.31**	**1.57**	**0.98**	**25.1**	**23.2**	**16.8**	**7.8**	**78.4**	**58.8**	**30.2**	**19.6**	**7.2**	**5.0**	**2.4**	**2.2**	**2.6**	**1.8**	**0.9**	**0.8**
**N6-methyladenine**																										
BglII	1DFM	m6A^GATCT	1.89	1.89	1.39	0.17	4.41	4.24	2.36	0.17	36.4	36.4	36.4	1.0	72.2	71.3	47.7	1.0	7.0	6.0	5.0	1.0	3.0	2.0	2.0	1.0
EcoRI	1CKQ	G^m6AATTC	2.07	2.07	0.55	0.58	6.02	5.44	1.17	0.58	26.4	26.4	10.8	8.8	87.7	78.8	18.1	8.8	7.0	6.0	3.0	1.0	2.0	1.0	1.0	1.0
EcoRV	1B94	GAT^m6ATC	1.42	1.32	0.50	1.42	4.83	1.82	0.50	3.02	35.1	35.1	9.2	17.1	87.3	44.3	9.2	43.0	6.5	2.0	1.0	4.5	3.0	1.0	1.0	2.0
PacI	3LDY	TTAAT^Tm6AA	1.90	1.90	0.26	0.39	5.31	4.36	0.26	0.95	24.6	24.6	2.9	5.6	77.4	66.4	2.9	11.0	8.0	4.0	1.0	4.0	2.0	1.0	1.0	1.0
PvuII	1PVI	Cm6AG^CTG	1.86	1.86	0.38	0.02	5.05	5.03	0.45	0.02	23.5	23.5	6.3	0.0	72.3	72.2	6.7	0.0	8.5	8.0	1.5	0.5	3.5	3.0	1.5	0.5
SgrAI	3DVO	Cm6A^CCGGYG	1.12	1.12	0.00	0.08	1.56	1.48	0.00	0.08	29.6	29.6	0.0	0.3	34.0	33.7	0.0	0.3	3.0	2.0	0.0	1.0	2.0	1.0	0.0	1.0
		**Average:**	**1.71**	**1.69**	**0.51**	**0.44**	**4.53**	**3.73**	**0.79**	**0.80**	**29.3**	**29.3**	**10.9**	**5.5**	**71.8**	**61.1**	**14.1**	**10.7**	**6.7**	**4.7**	**1.9**	**2.0**	**2.6**	**1.5**	**1.1**	**1.1**
**N4-methylcytosine**																										
BcnI	2ODI	CC^m4CGG	2.13	2.13	0.00	0.25	5.24	4.94	0.00	0.30	25.8	25.8	0.0	2.0	75.7	73.6	0.0	2.1	8.0	6.0	0.0	2.0	3.0	2.0	0.0	1.0
BglI	1DMU	GCm4CN$_4$^NGGC	0.49	0.49	0.49	0.00	1.13	1.13	1.13	0.00	8.8	8.8	8.8	0.0	14.7	14.7	14.7	0.0	3.0	3.0	3.0	0.0	3.0	3.0	3.0	0.0
BglI	1DMU	Gm4CCN$_4$^NGGC	1.81	1.81	1.81	0.51	6.97	6.46	6.04	0.51	39.5	39.5	39.5	7.3	129.6	122.3	115.4	7.3	7.0	6.0	5.0	1.0	3.0	2.0	2.0	1.0
BsoBI	1DC1	m4C^YCGRG	2.00	2.00	2.00	0.00	5.41	5.41	5.41	0.00	25.2	25.2	25.2	0.0	78.7	78.7	78.7	0.0	6.0	6.0	6.0	0.0	2.0	2.0	2.0	0.0
BsoBI	1DC1	C^Ym4CGRG	1.85	1.85	0.00	0.65	4.95	4.30	0.00	0.65	27.5	27.5	0.0	14.2	86.7	72.5	0.0	14.2	5.0	4.0	0.0	1.0	2.0	1.0	0.0	1.0
HindIII	2E52	A^AGm4CTT	1.81	1.81	0.28	0.00	5.20	5.20	0.28	0.00	26.0	26.0	3.2	0.0	80.0	80.0	3.2	0.0	5.0	5.0	1.0	0.0	2.0	2.0	1.0	0.0
MspI	1SA3	m4C^CGG	1.35	1.35	1.14	0.04	3.03	2.95	1.33	0.08	28.6	28.6	28.6	0.1	58.6	58.4	30.2	0.2	4.8	3.5	2.0	1.3	2.0	1.5	1.0	0.5
MvaI	2OAA	m4CC^WGG	1.81	1.81	1.41	0.24	5.52	5.28	3.94	0.24	31.3	31.3	31.3	4.4	96.1	91.7	73.4	4.4	6.5	6.0	5.0	0.5	2.5	2.0	2.0	0.5
NaeI	1IAW	GCm5C^GGC	1.06	0.80	0.00	1.00	6.51	1.97	0.00	4.54	26.1	14.4	0.0	25.6	134.2	30.7	0.0	103.5	11.3	4.0	0.0	7.3	3.0	2.0	0.0	1.0
SfiI	2EZV	GGCm4CN$_4$^NGGCC	1.69	1.69	0.48	0.00	5.06	5.06	0.68	0.00	25.6	25.6	8.8	0.0	75.0	75.0	10.2	0.0	6.0	6.0	2.0	0.0	2.0	2.0	1.0	0.0
		**Average:**	**1.66**	**1.66**	**0.84**	**0.19**	**4.72**	**4.52**	**2.09**	**0.20**	**26.5**	**26.5**	**16.2**	**3.1**	**77.2**	**74.1**	**36.2**	**3.1**	**5.7**	**5.1**	**2.7**	**0.6**	**2.4**	**1.9**	**1.3**	**0.4**
		**Average of all 3 modif.:**	**1.37**	**1.35**	**0.71**	**0.34**	**4.44**	**3.69**	**1.57**	**0.75**	**26.1**	**25.1**	**15.6**	**6.2**	**77.0**	**63.1**	**29.0**	**13.8**	**6.7**	**4.9**	**2.4**	**1.8**	**2.5**	**1.8**	**1.0**	**0.7**

Table S3. Statistical analysis of the clashes between the *in silico* introduced methyl groups and protein/DNA atoms. Shapiro-Wilk test for normality (H0 = data drawn from normal distribution); Wilcoxon rank sum test (two-sided, H0 = two sets of measurements are drawn from the same distribution); ROC (Receiver Operating Characteristic); prot – protein; mc – main chain.

A. All types of methylation analyzed together

			Most drastic clash distance				Sum of clash distances				Most drastic overlap volume				Sum of overlap volumes				Number of contacts				No of contacted residues			
Test	methylation		prot+ DNA	prot	prot mc	DNA	prot+ DNA	prot	prot mc	DNA	prot+ DNA	prot	prot mc	DNA	prot+ DNA	prot	prot mc	DNA	prot+ DNA	prot	prot mc	DNA	prot+ DNA	prot	prot mc	DNA
Shapiro-Wilk	cognate	p value	0.023	0.027	0.033	0.106	2.1E-3	3.0E-3	1.8E-3	1.6E-3	0.047	0.026	0.020	0.001	0.095	4.8E-3	1.4E-3	9.3E-5	0.120	0.029	0.035	0.037	0.065	0.021	6.6E-3	7.7E-4
		is distribution normal?	N	N	N	Y	N	N	N	N	N	N	N	N	Y	N	N	N	Y	N	N	N	Y	N	N	N
	tolerated	p value	0.104	0.033	7.1E-5	2.9E-4	0.011	0.008	5.9E-7	1.6E-6	0.297	0.118	1.1E-5	7.2E-5	0.024	7.2E-3	1.0E-6	7.4E-7	0.072	0.126	2.8E-6	9.8E-4	0.415	0.062	6.5E-4	1.7E-4
		is distribution normal?	Y	N	N	N	N	N	N	N	Y	Y	N	N	N	N	N	N	Y	Y	N	N	Y	Y	N	N
	protective	p value	0.347	0.307	0.021	4.1E-4	0.591	0.175	1.9E-4	1.6E-8	0.065	0.059	3.0E-3	1.7E-5	0.059	0.475	2.8E-4	2.0E-9	0.012	0.047	1.0E-3	1.4E-5	2.4E-5	1.9E-4	4.0E-4	5.4E-5
		is distribution normal?	Y	Y	N	N	Y	Y	N	N	Y	Y	N	N	Y	Y	N	N	N	N	N	N	N	N	N	N
Wilcoxon rank sum	cognate vs tolerated	p value	5.0E-5	1.2E-5	1.8E-2	0.752	2.4E-4	7.0E-6	0.020	0.846	1.5E-4	1.5E-5	0.025	0.697	1.4E-4	3.9E-6	0.017	0.884	0.012	2.4E-5	0.025	0.706	1.4E-3	9.1E-4	0.036	0.627
		are sets significantly different?	Y	Y	Y	N	Y	Y	Y	N	Y	Y	Y	N	Y	Y	Y	N	Y	Y	Y	N	Y	Y	Y	N
Wilcoxon rank sum	protective vs tolerated	p value	5.9E-5	1.2E-5	0.005	0.812	1.2E-4	6.8E-6	8.1E-3	0.883	1.3E-5	2.0E-6	4.7E-3	0.896	6.3E-5	3.8E-6	7.7E-3	0.909	6.8E-3	1.1E-4	0.018	0.954	0.016	8.7E-3	0.098	0.948
		are sets significantly different?	Y	Y	Y	N	Y	Y	Y	N	Y	Y	Y	N	Y	Y	Y	N	Y	Y	Y	N	Y	Y	N	N
Wilcoxon rank sum	cognate vs protective	p value	0.134	0.119	0.993	0.953	0.703	0.498	0.980	0.919	0.253	0.365	0.537	0.966	0.993	0.537	0.939	0.993	0.648	0.488	0.866	0.678	0.046	0.106	0.426	0.520
		are sets significantly different?	N	N	N	N	N	N	N	N	N	N	N	N	N	N	N	N	N	N	N	N	Y	N	N	N
ROC	cognate vs tolerated	Area under the curve	0.86	0.89	0.71	0.53	0.83	0.90	0.71	0.52	0.84	0.89	0.70	0.53	0.84	0.91	0.71	0.51	0.73	0.88	0.70	0.53	0.78	0.80	0.69	0.54

B. C5-methylcytosine

			Most drastic clash distance				Sum of clash distances				Most drastic overlap volume				Sum of overlap volumes				Number of contacts				No of contacted residues			
Test	methylation		prot+ DNA	prot	prot mc	DNA	prot+ DNA	prot	prot mc	DNA	prot+ DNA	prot	prot mc	DNA	prot+ DNA	prot	prot mc	DNA	prot+ DNA	prot	prot mc	DNA	prot+ DNA	prot	prot mc	DNA
Shapiro-Wilk	cognate	p value	0.12	0.25	0.79	0.09	0.45	0.09	0.37	0.10	0.96	0.50	0.67	0.03	0.70	0.25	0.36	0.10	0.57	0.60	0.33	0.50	0.01	0.31	0.97	0.00
		is distribution normal?	Y	Y	Y	Y	Y	Y	Y	Y	Y	Y	Y	N	Y	Y	Y	Y	Y	Y	Y	Y	N	Y	Y	N
	tolerated	p value	0.47	0.58	0.00	0.17	0.30	0.20	0.00	0.04	0.03	0.19	0.00	0.03	0.41	0.06	0.00	0.02	0.31	0.40	0.00	0.07	0.40	0.41	0.02	0.00
		is distribution normal?	Y	Y	N	Y	Y	Y	N	N	N	Y	N	N	Y	Y	N	N	Y	Y	N	Y	Y	Y	N	N
	protective	p value	0.31	0.39	0.01	0.05	0.53	0.93	0.01	0.00	0.55	0.57	0.03	0.00	0.19	0.83	0.02	0.00	0.06	0.27	0.01	0.00	0.00	0.01	0.00	0.00
		is distribution normal?	Y	Y	N	Y	Y	Y	N	N	Y	Y	N	N	Y	Y	N	N	Y	Y	N	N	N	N	N	N
Wilcoxon rank sum	cognate vs tolerated	p value	0.042	0.021	0.431	0.309	0.021	0.033	0.431	0.165	0.021	0.010	0.331	0.309	0.042	0.016	0.287	0.267	0.087	0.151	0.517	0.079	0.309	0.643	0.547	0.165
		are sets significantly different?	Y	Y	N	N	Y	Y	N	N	Y	Y	N	N	Y	Y	N	N	N	N	N	N	N	N	N	N
Wilcoxon rank sum	protective vs tolerated	p value	2.5E-3	2.5E-3	0.059	0.903	2.1E-3	2.2E-3	0.124	0.972	1.1E-3	8.9E-4	0.059	0.889	2.3E-3	2.3E-3	0.086	0.972	0.024	1.0E-2	0.248	0.972	0.195	0.162	0.713	0.766
		are sets significantly different?	Y	Y	N	N	Y	Y	N	N	Y	Y	N	N	Y	Y	N	N	Y	Y	N	N	N	N	N	N
Wilcoxon rank sum	cognate vs protective	p value	0.839	0.786	0.684	0.277	0.946	0.839	0.684	0.135	0.684	0.892	0.684	0.342	0.892	0.892	0.684	0.221	0.610	0.435	0.760	0.067	0.684	0.415	0.684	0.118
		are sets significantly different?	N	N	N	N	N	N	N	N	N	N	N	N	N	N	N	N	N	N	N	N	N	N	N	N

C. N6-methyladenine

			Most drastic clash distance				Sum of clash distances				Most drastic overlap volume				Sum of overlap volumes				Number of contacts				No of contacted residues			
Test	methylation		prot+ DNA	prot	prot mc	DNA	prot+ DNA	prot	prot mc	DNA	prot+ DNA	prot	prot mc	DNA	prot+ DNA	prot	prot mc	DNA	prot+ DNA	prot	prot mc	DNA	prot+ DNA	prot	prot mc	DNA
Shapiro-Wilk	cognate	p value	0.13	0.13	0.01	0.21	0.86	0.50	0.00	0.11	0.20	0.20	0.00	0.24	0.12	0.10	0.00	0.24	0.58	0.59	0.08	0.39	0.07	0.00	0.05	0.01
		is distribution normal?	Y	Y	N	Y	Y	Y	N	Y	Y	Y	N	Y	Y	Y	N	Y	Y	Y	Y	Y	Y	N	Y	N
	tolerated	p value	0.39	0.10	0.00	0.00	0.01	0.01	0.00	0.00	0.53	0.41	0.00	0.00	0.03	0.08	0.00	0.00	0.13	0.49	0.01	0.00	0.74	0.08	0.01	0.03
		is distribution normal?	Y	Y	N	N	N	N	N	N	Y	Y	N	N	N	Y	N	N	Y	Y	N	N	Y	Y	N	N
	protective	p value	0.19	0.13	0.23	0.10	0.09	0.17	0.16	0.02	0.34	0.34	0.04	0.16	0.04	0.22	0.06	0.01	0.11	0.42	0.38	0.02	0.07	0.01	0.51	0.03
		is distribution normal?	Y	Y	Y	Y	Y	Y	Y	N	Y	Y	N	Y	N	Y	Y	N	Y	Y	Y	N	Y	N	Y	N
Wilcoxon rank sum	cognate vs tolerated	p value	0.028	0.015	0.524	0.355	0.105	7.8E-3	0.452	0.563	0.064	0.011	0.488	0.355	0.037	3.8E-3	0.418	0.563	0.093	1.2E-3	0.452	0.729	0.013	3.2E-3	0.563	0.817
		are sets significantly different?	Y	Y	N	N	N	Y	N	N	N	Y	N	N	Y	Y	N	N	N	Y	N	N	Y	Y	N	N
Wilcoxon rank sum	protective vs tolerated	p value	0.039	0.020	0.033	0.156	0.156	0.020	0.028	0.245	0.039	6.7E-3	0.039	0.121	0.071	6.7E-3	0.039	0.197	0.197	0.053	0.053	0.366	0.045	0.220	0.081	0.220
		are sets significantly different?	Y	Y	Y	N	N	Y	Y	N	Y	Y	Y	N	N	Y	Y	N	N	N	N	N	Y	N	N	N
Wilcoxon rank sum	cognate vs protective	p value	0.886	0.775	0.225	0.568	0.886	0.668	0.432	0.317	0.568	0.568	0.225	0.475	0.568	0.199	0.284	0.317	0.475	0.317	0.432	0.353	0.520	0.086	0.475	0.199
		are sets significantly different?	N	N	N	N	N	N	N	N	N	N	N	N	N	N	N	N	N	N	N	N	N	N	N	N

D. N4-methylcytosine

			Most drastic clash distance				Sum of clash distances				Most drastic overlap volume				Sum of overlap volumes				Number of contacts				No of contacted residues			
Test	methylation		prot+ DNA	prot	prot mc	DNA	prot+ DNA	prot	prot mc	DNA	prot+ DNA	prot	prot mc	DNA	prot+ DNA	prot	prot mc	DNA	prot+ DNA	prot	prot mc	DNA	prot+ DNA	prot	prot mc	DNA
Shapiro-Wilk	cognate	p value	0.01	0.01	0.56	0.02	0.03	0.02	0.21	0.01	0.03	0.03	0.96	0.00	0.06	0.01	0.19	0.00	0.02	0.03	0.19	0.69	0.48	0.27	0.00	0.06
		is distribution normal?	N	N	Y	N	N	N	Y	N	N	N	Y	N	Y	N	Y	N	N	N	Y	Y	Y	Y	N	Y
	protective	p value	0.01	0.01	0.24	0.02	0.06	0.10	0.03	0.03	0.06	0.06	0.17	0.00	0.34	0.23	0.05	0.00	0.92	0.01	0.33	0.08	0.00	0.02	0.36	0.01
		is distribution normal?	N	N	Y	N	Y	Y	N	N	Y	Y	Y	N	Y	Y	Y	N	Y	N	Y	Y	N	N	Y	N
Wilcoxon rank-sum	cognate vs protective	p value	0.825	0.825	0.453	0.566	0.627	0.402	0.566	0.508	0.122	0.122	0.757	0.627	0.566	0.627	0.453	0.566	0.093	0.354	0.377	0.158	0.064	0.270	0.596	0.233
		are sets significantly different?	N	N	N	N	N	N	N	N	N	N	N	N	N	N	N	N	N	N	N	N	N	N	N	N

Table S4. Statistical analysis of the influence of the adaptive fit and coordinate errors on the discriminative properties of analyzed clash measures. Sums of van der Waals radii of contacting atoms have been reduced by 0.1, 0.2, 0.3 and 0.4 Å and the p-values of the studied clash measures were calculated using Wilcoxon rank sum test (two-sided, H0 = two sets of measurements are drawn from the same distribution); prot – protein; mc – main chain. The areas under the Receiver Operating Characteristic (ROC) curves were calculated using cognate protective and non-cognate tolerated cases.

Sum of van der Waals radii reduced by [Å]:	Most drastic clash distance				Sum of clash distances				Most drastic overlap volume				Sum of overlap volumes				Number of contacts				No of contacted residues			
	prot+ DNA	prot	prot mc	DNA	prot+ DNA	prot	prot mc	DNA	prot+ DNA	prot	prot mc	DNA	prot+ DNA	prot	prot mc	DNA	prot+ DNA	prot	prot mc	DNA	prot+ DNA	prot	prot mc	DNA
**cognate vs tolerated**																								
0.1	5.0E-5	1.2E-5	0.018	0.750	2.4E-4	7.0E-6	0.020	0.850	1.5E-4	1.5E-5	0.025	0.700	1.4E-4	3.9E-6	0.017	0.880	0.012	2.4E-5	0.025	0.710	1.4E-3	9.1E-4	0.036	0.630
0.2	5.0E-5	1.2E-5	0.019	0.680	2.4E-4	7.8E-6	0.022	0.840	6.1E-5	4.4E-6	0.028	0.690	6.8E-5	3.5E-6	0.017	0.870	0.011	2.6E-5	0.024	0.990	2.3E-3	4.9E-4	0.034	0.670
0.3	5.0E-5	1.2E-5	0.020	0.620	1.8E-4	4.9E-6	0.022	0.730	3.6E-5	2.7E-6	0.029	0.510	7.5E-5	3.5E-6	0.017	0.640	4.8E-3	4.0E-5	0.029	0.790	3.0E-3	1.0E-3	0.042	0.470
0.4	5.0E-5	1.2E-5	0.025	0.650	8.3E-5	7.0E-6	0.026	0.740	5.0E-5	4.9E-6	0.029	0.780	5.0E-5	5.5E-6	0.021	0.830	7.7E-4	2.5E-5	0.022	0.720	0.016	3.4E-3	0.053	0.660
**cognate vs non-cog. protective**																								
0.1	0.130	0.120	0.990	0.950	0.700	0.500	0.980	0.920	0.250	0.370	0.540	0.970	0.990	0.540	0.940	0.990	0.650	0.490	0.870	0.680	0.046	0.110	0.430	0.520
0.2	0.130	0.120	0.940	0.880	0.720	0.480	0.920	0.920	0.290	0.420	0.520	0.910	0.960	0.560	0.920	0.930	0.810	0.920	0.840	0.760	0.160	0.100	0.860	0.720
0.3	0.130	0.120	0.930	0.760	0.790	0.440	0.930	0.770	0.940	0.810	0.500	0.780	0.980	0.700	0.890	0.850	0.610	0.370	0.840	0.640	0.360	0.230	0.890	0.790
0.4	0.130	0.120	0.970	0.980	0.750	0.430	0.930	0.980	0.850	0.790	0.610	0.940	0.890	0.860	0.990	0.890	0.680	0.290	0.890	0.970	0.820	0.720	0.830	0.950
**non-cog protective vs non-cog. tolerated**																								
0.1	5.9E-5	1.2E-5	5.3E-3	0.810	1.2E-4	6.8E-6	8.1E-3	0.880	1.3E-5	2.0E-6	4.7E-3	0.900	6.3E-5	3.8E-6	7.7E-3	0.910	6.8E-3	1.1E-4	1.8E-2	0.950	0.016	8.7E-3	0.098	0.950
0.2	5.9E-5	1.3E-5	3.6E-3	0.840	8.9E-5	8.6E-6	4.8E-3	0.980	9.6E-6	1.8E-6	3.0E-3	0.930	3.1E-5	3.2E-6	5.1E-3	0.970	4.3E-3	7.3E-5	6.0E-3	0.750	0.018	6.5E-3	0.019	0.920
0.3	5.9E-5	1.3E-5	2.6E-3	0.930	6.3E-5	7.1E-6	3.7E-3	0.990	1.2E-5	2.3E-6	2.4E-3	0.760	1.6E-5	3.2E-6	3.6E-3	0.790	2.9E-3	1.4E-4	5.6E-3	0.850	5.7E-3	5.1E-3	0.014	0.740
0.4	5.9E-5	1.4E-5	4.9E-3	0.670	5.3E-5	6.6E-6	7.0E-3	0.720	1.8E-5	2.8E-6	5.2E-3	0.760	9.6E-6	3.2E-6	6.7E-3	0.740	2.7E-4	3.6E-5	8.3E-3	0.680	4.5E-3	2.2E-3	0.029	0.610
**m6A cognate vs tolerated**																								
0.1	0.028	0.015	0.520	0.350	0.110	7.8E-3	0.450	0.560	0.064	0.011	0.490	0.350	0.037	3.8E-3	0.420	0.560	0.093	1.2E-3	0.450	0.730	0.013	3.2E-3	0.560	0.820
0.2	0.028	0.015	0.560	0.300	0.110	0.011	0.520	0.490	0.028	3.8E-3	0.560	0.300	0.037	3.8E-3	0.560	0.420	0.064	1.5E-3	0.490	0.600	0.015	3.2E-3	0.520	0.490
0.3	0.028	0.015	0.420	0.450	0.110	0.011	0.420	0.600	0.011	1.2E-3	0.420	0.350	0.037	3.8E-3	0.420	0.350	0.110	1.8E-3	0.390	0.690	0.015	3.8E-3	0.420	0.520
0.4	0.028	0.015	0.490	0.560	0.083	0.011	0.490	0.560	0.021	2.6E-3	0.490	0.560	0.037	7.8E-3	0.490	0.560	0.056	2.6E-3	0.420	0.690	0.056	6.5E-3	0.450	0.690
**m5C cognate vs tolerated**																								
0.1	0.042	0.021	0.430	0.310	0.021	0.033	0.430	0.160	0.021	0.010	0.330	0.310	0.042	0.016	0.290	0.270	0.087	0.150	0.520	0.079	0.310	0.640	0.550	0.160
0.2	0.042	0.021	0.400	0.310	0.033	0.026	0.400	0.160	0.016	7.3E-3	0.310	0.230	0.026	0.010	0.270	0.310	0.079	0.130	0.430	0.064	0.180	0.460	0.490	0.120
0.3	0.042	0.021	0.380	0.270	0.033	0.012	0.330	0.230	0.016	0.010	0.290	0.230	0.026	0.012	0.250	0.310	0.064	0.079	0.380	0.190	0.190	0.380	0.400	0.250
0.4	0.042	0.021	0.380	0.230	0.033	0.016	0.290	0.310	0.016	0.012	0.330	0.230	0.026	0.012	0.330	0.230	0.064	0.052	0.250	0.190	0.150	0.350	0.330	0.330
**ROC (Receiver Operating Characteristic) - area under the curve**																								
0.1	0.855	0.886	0.725	0.585	0.829	0.902	0.724	0.550	0.852	0.898	0.719	0.581	0.848	0.910	0.721	0.555	0.738	0.875	0.695	0.543	0.788	0.818	0.680	0.595
0.2	0.862	0.890	0.709	0.537	0.827	0.898	0.704	0.518	0.857	0.909	0.696	0.536	0.855	0.913	0.713	0.514	0.727	0.875	0.701	0.501	0.772	0.811	0.689	0.538
0.3	0.862	0.890	0.708	0.544	0.833	0.907	0.704	0.530	0.868	0.918	0.695	0.558	0.853	0.913	0.712	0.541	0.751	0.866	0.695	0.524	0.764	0.792	0.681	0.565
0.4	0.862	0.890	0.700	0.540	0.851	0.900	0.698	0.529	0.862	0.907	0.695	0.525	0.862	0.905	0.706	0.520	0.800	0.876	0.705	0.533	0.715	0.761	0.672	0.539

Table S5. Methyl groups ordered according to the severity of steric conflict. For each enzyme, methyl groups were grafted separately onto all possible target DNA bases, and clashes were scored. Results for cognate protective, non-cognate protective and tolerated methyl groups are presented on dark blue, light blue and light orange background, respectively.

Methylation type	methylated sequence	effect of methylation	Most drastic clash distance (Å)				Sum of clash distances (Å)				Most drastic overlap volume ($Å^3$)				Sum of overlap volumes ($Å^3$)				Number of contacts				No of contacted residues			
			prot+ DNA	prot	prot mc	DNA	prot+ DNA	prot	prot mc	DNA	prot+ DNA	prot	prot mc	DNA	prot+ DNA	prot	prot mc	DNA	prot+ DNA	prot	prot mc	DNA	prot+ DNA	prot	prot mc	DNA
**BamHI (2BAM)**																										
m4C	G^GATCm4C	no data	1.86	1.86	0.56	0.00	5.53	5.53	0.56	0.00	28.36	28.36	11.14	0.00	91.98	91.98	11.14	0.00	6	6	1	0	2	2	1	0
**m4C**	**G^GATm4CC**	**cog. protective**	**1.75**	**1.75**	**1.75**	**0.69**	**6.37**	**5.01**	**4.58**	**1.37**	**27.05**	**26.27**	**26.27**	**15.39**	**104.11**	**80.69**	**72.64**	**23.42**	**11**	**7.5**	**6**	**3.5**	**3.5**	**2.5**	**2**	**1**
m5C	G^GATm5CC	ncog. protective	1.05	1.05	1.05	0.41	5.51	4.88	4.20	0.63	25.23	25.23	25.23	6.52	106.08	97.77	82.49	8.32	8	6	5	2	3	2	1	1
m5C	G^GATCm5C	ncog. tolerated	1.04	1.04	0.10	0.51	2.85	1.32	0.10	1.54	15.58	15.58	0.89	9.59	40.82	17.85	0.89	22.98	7.5	2.5	0.5	5	2	1	0.5	1
m6A	G^Gm6ATCC	ncog. tolerated	0.07	0.06	0.06	0.02	0.08	0.06	0.06	0.02	0.33	0.32	0.32	0.02	0.34	0.32	0.32	0.02	1.5	0.5	0.5	1	1.5	0.5	0.5	1
**BcnI (2ODI)**																										
m4C	CC^m4CGG	ncog. protective	2.13	2.13	0.00	0.25	5.24	4.94	0.00	0.30	25.78	25.78	0.00	2.01	75.69	73.58	0.00	2.11	8	6	0	2	3	2	0	1
**m4C**	**Cm4C^SGG**	**cog. protective**	**1.82**	**1.82**	**1.29**	**0.15**	**4.58**	**4.38**	**2.61**	**0.20**	**21.51**	**21.51**	**18.54**	**1.37**	**68.36**	**66.76**	**43.32**	**1.60**	**6.75**	**5.5**	**2.75**	**1.25**	**3.5**	**2.75**	**1.25**	**0.75**
m4C	m4CC^SGG	no data	1.73	1.73	1.24	0.08	5.18	5.11	3.90	0.08	29.69	29.69	29.69	0.61	86.12	85.51	72.62	0.61	7.5	7	5	0.5	3	2.5	2	0.5
m5C	CC^m5CGG	no data	1.57	1.57	0.00	0.38	4.14	3.00	0.00	1.15	27.53	27.53	0.00	5.75	65.67	52.10	0.00	13.57	8	3	0	5	2	1	0	1
m5C	Cm5C^SGG	no data	0.93	0.41	0.30	0.84	4.85	1.42	0.40	3.43	21.37	6.67	5.19	20.21	92.76	20.63	5.66	72.14	9.75	3.75	1.5	6	2.5	1.5	1.5	1
m5C	m5CC^SGG	ncog. tolerated	0.93	0.93	0.91	0.17	2.62	2.44	2.04	0.18	20.51	20.51	20.36	1.11	44.96	43.82	37.42	1.14	5.75	4.5	4	1.25	3	2	2	1
**BfiI (3ZI5)**																										
m6A	5' m6ACTGGG 3'	no data	2.22	2.22	1.57	0.35	6.69	6.18	3.66	0.51	39.51	39.51	39.51	2.89	112.74	108.81	84.71	3.94	7.5	5.5	3.5	2	3.5	1.5	1.5	2
**m4C**	**3' TGACm4CC 5'**	**cog. protective**	**2.06**	**2.06**	**0.55**	**0.00**	**6.00**	**6.00**	**0.55**	**0.00**	**26.21**	**26.21**	**10.83**	**0.00**	**84.76**	**84.76**	**10.83**	**0.00**	**6.25**	**6.25**	**1.25**	**0**	**2.5**	**2.5**	**1**	**0**
m4C	3' TGACCm4C 5'	no data	1.84	1.84	1.84	0.21	5.42	5.21	5.16	0.21	29.94	29.94	29.94	1.44	96.72	95.28	95.09	1.44	5.5	4.5	4	1	2	1	1	1
m4C	3' TGAm4CCC 5'	no data	1.31	1.31	0.00	0.31	4.94	4.56	0.00	0.38	21.40	21.40	0.00	3.07	76.45	73.16	0.00	3.29	8	6	0	2	4	3	0	1
m5C	3' TGACCm5C 5'	no data	1.19	1.19	1.19	0.10	4.62	4.52	4.21	0.10	15.40	15.40	15.40	0.84	62.01	61.17	58.53	0.84	9.5	9	8	0.5	3.5	3	3	0.5
m5C	3' TGAm5CCC 5'	no data	1.03	1.03	0.00	0.65	4.97	3.27	0.00	1.70	19.67	19.67	0.00	13.91	85.10	54.98	0.00	30.12	10	5.5	0	4.5	2	1	0	1
m5C	3' TGACm5CC 5'	no data	1.02	1.00	0.00	0.39	3.25	1.59	0.00	1.66	18.51	15.98	0.00	8.48	53.14	22.44	0.00	30.70	8	3.25	0	4.75	2.75	1.75	0	1
m6A	3' TGm6ACCC 5'	no data	0.30	0.01	0.00	0.30	0.46	0.01	0.00	0.46	3.04	0.01	0.00	3.04	4.01	0.01	0.00	4.00	3	0.5	0	2.5	1.5	0.5	0	1
**BglII (1DFM)**																										
**m4C**	**A^GATm4CT**	**cog. protective**	**1.89**	**1.89**	**1.89**	**0.00**	**5.25**	**5.25**	**5.25**	**0.00**	**23.00**	**23.00**	**23.00**	**0.00**	**80.89**	**80.89**	**80.89**	**0.00**	**7**	**7**	**7**	**0**	**2**	**2**	**2**	**0**
m6A	m6A^GATCT	ncog. protective	1.89	1.89	1.39	0.17	4.41	4.24	2.36	0.17	36.45	36.45	36.45	0.97	72.23	71.27	47.66	0.97	7	6	5	1	3	2	2	1
m6A	A^Gm6ATCT	ncog. tolerated	0.85	0.85	0.00	0.00	2.11	2.11	0.00	0.00	13.93	13.93	0.00	0.00	30.46	30.46	0.00	0.00	4	4	0	0	2	2	0	0
m5C	A^GATm5CT	ncog. protective	0.60	0.60	0.60	0.57	2.33	1.62	1.62	0.71	11.17	9.48	9.48	11.17	34.05	21.87	21.87	12.18	7.5	5.5	5.5	2	3	2	2	1
**BstYI (1VRR)**																										
**m4C**	**R^GATm4CY**	**cog. protective**	**1.22**	**1.22**	**1.22**	**0.05**	**3.83**	**3.77**	**3.77**	**0.06**	**20.68**	**20.68**	**20.68**	**0.07**	**64.06**	**63.99**	**63.99**	**0.08**	**7**	**5.5**	**5.5**	**1.5**	**3**	**2**	**2**	**1**
m5C	R^GATm5CY	no data	0.83	0.33	0.33	0.83	2.10	0.75	0.75	1.36	20.02	2.61	2.61	20.02	32.23	5.60	5.60	26.63	7.5	4.5	4.5	3	2	1	1	1
m6A	R^Gm6ATCY	ncog. tolerated	0.51	0.51	0.25	0.00	0.99	0.99	0.25	0.00	9.40	9.40	3.41	0.00	16.34	16.34	3.41	0.00	3	3	1	0	1	1	1	0
**Ecl18kI (2FQZ)**																										
m4C	^Cm4CNGG	no data	2.06	2.06	0.00	0.41	5.61	5.21	0.00	0.41	27.09	27.09	0.00	4.98	73.74	68.76	0.00	4.98	6	5	0	1	3	2	0	1
m5C	^m5CCNGG	no data	1.48	1.48	0.12	0.74	6.27	4.43	0.12	1.84	25.64	25.64	0.86	17.05	108.04	74.73	0.86	33.31	10	5	1	5	3	1	1	2
m4C	^m4CCNGG	no data	1.32	1.32	1.28	0.32	4.19	3.88	1.32	0.32	33.89	33.89	33.89	3.32	78.86	75.54	34.02	3.32	6.75	5.75	1.75	1	3	2	1.25	1
**m5C**	**^Cm5CNGG**	**cog. protective**	**0.69**	**0.69**	**0.00**	**0.35**	**1.82**	**1.21**	**0.00**	**0.62**	**15.42**	**15.42**	**0.00**	**5.08**	**29.14**	**21.46**	**0.00**	**7.69**	**4.25**	**2**	**0**	**2.25**	**3**	**2**	**0**	**1**
**EcoO109I (1WTE)**																										

m4C	RG^GNCm4CY	no data	1.57	1.57	1.57	0.19	3.69	3.25	3.25	0.44	17.31	17.31	17.31	1.61	48.14	44.61	44.61	3.53	7	4.5	4.5	2.5	2.5	1.5	1.5	1
m4C	RG^GNm4CCY	no data	1.51	1.51	1.51	0.78	4.18	2.63	2.63	1.56	17.24	17.24	17.24	13.57	57.54	34.59	34.59	22.95	7	4	4	3	3	2	2	1
m5C	RG^GNCm5CY	ncog. protective	1.43	1.34	1.34	1.11	9.16	3.32	2.95	5.85	37.31	35.14	35.14	28.33	194.47	63.02	59.18	131.45	14.5	6	4	8.5	2	1	1	1
**m5C**	**RG^GNm5CCY**	**cog. protective**	**1.33**	**1.33**	**1.33**	**0.56**	**5.77**	**3.97**	**3.48**	**1.80**	**34.84**	**34.84**	**34.84**	**9.20**	**99.25**	**76.41**	**67.23**	**22.84**	**12**	**6**	**5**	**6**	**2.5**	**1.5**	**1.5**	**1**
m6A	m6AG^GNCCY	ncog. tolerated	0.94	0.94	0.00	0.00	0.94	0.94	0.00	0.00	24.10	24.10	0.00	0.00	24.10	24.10	0.00	0.00	1	1	0	0	1	1	0	0
m4C	RG^GNCCm4C	no data	0.41	0.33	0.00	0.41	0.74	0.33	0.00	0.41	4.95	3.38	0.00	4.95	8.33	3.38	0.00	4.95	2	1	0	1	2	1	0	1
m5C	RG^GNCCm5C	ncog. tolerated	0.22	0.22	0.00	0.16	0.38	0.22	0.00	0.16	2.22	2.22	0.00	1.23	3.45	2.22	0.00	1.23	2	1	0	1	2	1	0	1
**EcoRI (1CKQ)**																										
m6A	G^m6AATTC	ncog. protective	2.07	2.07	0.55	0.58	6.02	5.44	1.17	0.58	26.35	26.35	10.79	8.84	87.65	78.81	18.10	8.84	7	6	3	1	2	1	1	1
m4C	G^AATTm4C	no data	2.03	2.03	2.03	0.23	7.10	6.87	6.87	0.23	26.31	26.31	26.31	2.40	106.64	104.24	104.24	2.40	10	9	9	1	4	3	3	1
**m6A**	**G^Am6ATTC**	**cog. protective**	**1.29**	**1.29**	**0.00**	**0.42**	**4.76**	**4.34**	**0.00**	**0.42**	**30.67**	**30.67**	**0.00**	**5.23**	**87.69**	**82.46**	**0.00**	**5.23**	**6**	**5**	**0**	**1**	**3**	**2**	**0**	**1**
m5C	G^AATTm5C	ncog. protective	0.91	0.91	0.69	0.00	3.31	3.31	2.13	0.00	18.87	18.87	15.30	0.00	53.82	53.82	31.83	0.00	7	7	5	0	2	2	1	0
**EcoRV (1B94)**																										
**m6A**	**Gm6AT^ATC**	**cog. protective**	**1.95**	**1.95**	**0.02**	**0.00**	**4.21**	**4.21**	**0.02**	**0.00**	**23.19**	**23.19**	**0.04**	**0.00**	**63.08**	**63.08**	**0.04**	**0.00**	**4.5**	**4.5**	**0.5**	**0**	**2**	**2**	**0.5**	**0**
m4C	GAT^ATm4C	no data	1.87	1.87	1.87	0.00	5.44	5.44	5.18	0.00	22.05	22.05	22.05	0.00	88.56	88.56	85.62	0.00	7	7	6	0	3	3	2	0
m6A	GAT^m6ATC	ncog. protective	1.42	1.32	0.50	1.42	4.83	1.82	0.50	3.02	35.07	35.07	9.21	17.06	87.30	44.28	9.21	43.02	6.5	2	1	4.5	3	1	1	2
m5C	GAT^ATm5C	ncog. tolerated	0.71	0.71	0.05	0.22	0.97	0.76	0.05	0.22	15.85	15.85	0.09	2.13	18.06	15.93	0.09	2.13	3	2	1	1	3	2	1	1
**FokI (1FOK)**																										
**m6A**	**3' CCTm6AC 5'**	**cog. protective**	**2.23**	**2.23**	**0.00**	**0.27**	**5.25**	**4.75**	**0.00**	**0.50**	**24.25**	**24.25**	**0.00**	**3.13**	**62.93**	**57.99**	**0.00**	**4.94**	**9**	**7**	**0**	**2**	**3**	**2**	**0**	**1**
**m6A**	**5' GGm6ATG 3'**	**cog. protective**	**1.96**	**1.96**	**0.00**	**0.27**	**4.76**	**4.49**	**0.00**	**0.27**	**23.09**	**23.09**	**0.00**	**3.13**	**65.03**	**61.90**	**0.00**	**3.13**	**7**	**6**	**0**	**1**	**4**	**3**	**0**	**1**
m5C	3' CCTAm5C 5'	no data	1.64	1.64	0.00	0.17	7.97	7.72	0.00	0.25	27.89	27.89	0.00	1.07	142.93	141.65	0.00	1.28	10	7	0	3	2	1	0	1
m4C	3' Cm4CTAC 5'	no data	1.55	1.55	0.00	0.37	4.15	3.64	0.00	0.51	28.91	28.91	0.00	5.53	74.64	68.37	0.00	6.27	5	3	0	2	2	1	0	1
m5C	3' m5CCTAC 5'	no data	1.48	1.48	1.48	0.86	8.36	5.26	4.28	3.10	21.40	19.68	19.68	21.40	142.26	81.87	66.04	60.39	15	8	6	7	3	2	2	1
m4C	3' m4CCTAC 5'	no data	1.42	1.42	1.42	0.51	3.41	2.21	2.21	1.20	17.05	17.05	17.05	7.31	42.33	27.91	27.91	14.42	7	3	3	4	2	1	1	1
m4C	3' CCTAm4C 5'	no data	1.16	1.16	1.04	0.00	4.81	4.81	1.04	0.00	27.32	27.32	27.32	0.00	93.45	93.45	27.32	0.00	6	6	1	0	1	1	1	0
m5C	3' Cm5CTAC 5'	ncog. tolerated	0.76	0.67	0.67	0.76	2.55	1.51	1.14	1.04	17.82	8.02	8.02	17.82	36.89	17.91	12.29	18.98	8	4	3	4	3	2	1	1
**HincII (1TX3)**																										
**m6A**	**GTY^Rm6AC**	**cog. protective**	**1.80**	**1.80**	**0.44**	**0.03**	**5.44**	**5.40**	**0.58**	**0.03**	**26.85**	**26.85**	**7.60**	**0.16**	**83.28**	**83.12**	**8.61**	**0.16**	**6.5**	**6.25**	**2.25**	**0.25**	**3.25**	**3**	**2**	**0.25**
m4C	GTY^RAm4C	no data	1.63	1.63	1.63	0.00	4.55	4.55	4.02	0.00	22.43	22.43	22.43	0.00	69.62	69.62	63.17	0.00	7	7	5.5	0	3.5	3.5	2	0
m4C	GTm4C^RAC	no data	1.20	1.20	1.20	0.00	1.23	1.23	1.23	0.00	31.58	31.58	31.58	0.00	31.68	31.68	31.68	0.00	1.5	1.5	1.5	0	1	1	1	0
m5C	GTY^RAm5C	ncog. protective	1.11	1.11	1.11	0.05	4.25	4.21	4.07	0.05	29.07	29.07	29.07	0.20	73.50	73.30	72.23	0.20	8.75	8.25	7.25	0.5	3.5	3	3	0.5
m5C	GTm5C^RAC	ncog. tolerated	0.92	0.92	0.88	0.05	2.34	2.29	0.88	0.05	23.35	23.35	22.01	0.49	44.97	44.48	22.01	0.49	4.5	4.25	1	0.25	2.5	2.25	1	0.25
**HindIII (2E52)**																										
**m6A**	**m6A^AGCTT**	**cog. protective**	**1.96**	**1.96**	**0.35**	**0.61**	**4.74**	**4.13**	**0.46**	**0.61**	**23.12**	**23.12**	**5.11**	**9.62**	**68.38**	**58.75**	**5.77**	**9.62**	**6.25**	**5**	**2**	**1.25**	**3**	**2**	**2**	**1**
m4C	A^AGm4CTT	ncog. protective	1.81	1.81	0.28	0.00	5.20	5.20	0.28	0.00	26.00	26.00	3.25	0.00	80.02	80.02	3.25	0.00	5	5	1	0	2	2	1	0
m5C	A^AGm5CTT	ncog. protective	0.91	0.91	0.00	0.16	2.94	2.72	0.00	0.22	17.01	17.01	0.00	0.87	40.80	39.75	0.00	1.05	8.25	6.25	0	2	3.25	2.25	0	1
m6A	A^m6AGCTT	ncog. tolerated	0.44	0.44	0.00	0.19	0.88	0.69	0.00	0.19	7.46	7.46	0.00	1.28	11.57	10.30	0.00	1.28	3	2	0	1	2	1	0	1
**HinP1I (2FKC)**																										
m4C	G^CGm4C	no data	2.06	2.06	1.16	0.01	6.49	6.48	3.03	0.01	30.42	30.42	18.63	0.02	102.82	102.80	50.96	0.02	7.75	7.5	3.5	0.25	2.75	2.5	1.5	0.25
m4C	G^m4CGC	no data	1.80	1.80	0.92	0.01	4.06	4.06	1.44	0.01	20.16	20.16	9.23	0.00	54.20	54.19	17.68	0.00	6.75	6.5	2	0.25	3	2.75	1	0.25
**m5C**	**G^m5CGC**	**cog. protective**	**1.21**	**1.15**	**0.71**	**0.45**	**4.54**	**3.97**	**1.77**	**0.57**	**25.42**	**23.65**	**15.24**	**9.50**	**80.85**	**70.60**	**30.13**	**10.25**	**7.5**	**5.5**	**3.5**	**2**	**3**	**2**	**2**	**1**
m5C	G^CGm5C	ncog. protective	1.20	1.14	1.08	0.80	4.06	2.95	2.15	1.11	28.18	21.03	21.03	18.91	72.29	48.38	36.75	23.91	7.5	5	4	2.5	2	1	1	1

**Hpy188I (3OQG)**																										
m4C	Tm4CN^GA	no data	1.84	1.84	1.84	0.01	4.86	4.85	4.85	0.01	23.24	23.24	23.24	0.00	72.88	72.88	72.88	0.00	7	6	6	1	3	2	2	1
**m6A**	**TCN^Gm6A**	**cog. protective**	**1.83**	**1.83**	**1.26**	**0.00**	**4.86**	**4.86**	**2.42**	**0.00**	**33.20**	**33.20**	**33.20**	**0.00**	**91.09**	**91.09**	**58.63**	**0.00**	**6**	**6**	**4**	**0**	**2**	**2**	**1**	**0**
m5C	Tm5CN^GA	no data	0.96	0.89	0.89	0.79	2.78	1.42	1.41	1.36	18.93	11.60	11.60	18.93	41.12	15.96	15.95	25.17	8	4.5	4	3.5	2.5	1.5	1	1
**Hpy99I (3GOX)**																										
**m4C**	**CGWm4CG^**	**cog. protective**	**2.01**	**2.01**	**0.12**	**0.66**	**4.73**	**3.83**	**0.12**	**0.90**	**22.68**	**22.68**	**0.70**	**10.71**	**62.95**	**50.95**	**0.70**	**12.00**	**7.5**	**5**	**1**	**2.5**	**4.5**	**2**	**1**	**2.5**
m4C	m4CGWCG^	no data	1.93	1.93	1.93	0.14	5.84	5.60	3.93	0.25	21.15	21.15	20.30	0.94	75.81	74.11	46.83	1.70	12	11	7	1	2.5	2	2	0.5
m5C	CGWm5CG^	no data	1.03	1.03	0.00	0.21	2.87	2.66	0.00	0.21	17.57	17.57	0.00	1.12	42.75	41.63	0.00	1.12	5	4	0	1	3	2	0	1
m5C	m5CGWCG^	no data	0.45	0.45	0.41	0.08	1.38	1.23	0.41	0.15	5.18	5.18	3.71	0.40	13.35	12.59	3.71	0.76	5.5	4.5	1	1	2.5	2	1	0.5
**MspI (1SA3)**																										
**m5C**	**m5C^CGG**	**cog. protective**	**1.43**	**1.43**	**0.20**	**0.35**	**3.50**	**2.71**	**0.20**	**0.79**	**21.69**	**21.69**	**3.03**	**5.19**	**50.45**	**40.50**	**3.03**	**9.95**	**7.25**	**4**	**0.5**	**3.25**	**2.5**	**1.5**	**0.5**	**1**
m4C	m4C^CGG	ncog. protective	1.35	1.35	1.14	0.04	3.03	2.95	1.33	0.08	28.64	28.64	28.64	0.13	58.56	58.36	30.19	0.21	4.75	3.5	2	1.25	2	1.5	1	0.5
m4C	C^m4CGG	no data	1.04	1.04	0.76	0.09	1.57	1.49	1.06	0.09	12.47	12.47	8.61	0.57	18.88	18.31	13.30	0.57	3	2.25	1	0.75	2.5	1.75	0.5	0.75
m5C	C^m5CGG	ncog. tolerated	0.82	0.49	0.23	0.40	3.28	2.25	0.23	1.03	16.41	9.23	2.16	7.66	52.63	35.21	2.16	17.42	6.75	4	0.5	2.75	1.5	0.5	0.5	1
**MunI (1D02)**																										
m6A	C^m6AATTG	no data	1.87	1.87	0.39	0.37	4.92	4.52	0.57	0.41	24.87	24.87	5.96	4.30	72.28	67.94	7.20	4.34	7	5	2	2	3	1	1	2
m4C	m4C^AATTG	no data	1.72	1.72	1.72	0.00	6.13	6.13	5.21	0.00	30.94	30.94	30.94	0.00	116.66	116.66	95.08	0.00	8	8	6	0	2	2	2	0
**m6A**	**C^Am6ATTG**	**cog. protective**	**1.34**	**1.34**	**0.00**	**0.48**	**5.16**	**4.66**	**0.00**	**0.51**	**35.45**	**35.45**	**0.00**	**6.61**	**92.68**	**86.05**	**0.00**	**6.64**	**9**	**7**	**0**	**2**	**3**	**2**	**0**	**1**
m5C	m5C^AATTG	no data	0.58	0.58	0.58	0.34	1.16	0.58	0.58	0.58	6.41	6.41	6.41	4.74	13.04	6.41	6.41	6.64	3.5	1	1	2.5	2	1	1	1
**MvaI (2OAA)**																										
m4C	m4CC^WGG	ncog. protective	1.81	1.81	1.41	0.24	5.52	5.28	3.94	0.24	31.28	31.28	31.28	4.38	96.12	91.74	73.42	4.38	6.5	6	5	0.5	2.5	2	2	0.5
**m4C**	**Cm4C^WGG**	**cog. protective**	**1.78**	**1.78**	**1.01**	**0.15**	**4.77**	**4.62**	**2.49**	**0.15**	**22.95**	**22.95**	**12.54**	**1.51**	**70.48**	**68.98**	**38.35**	**1.51**	**6.75**	**6.25**	**2.75**	**0.5**	**3.5**	**3**	**1.25**	**0.5**
m5C	m5CC^WGG	no data	1.16	1.16	1.02	0.09	2.55	2.43	1.81	0.12	26.55	26.55	26.55	0.49	48.91	48.30	37.91	0.61	4.75	3.5	3	1.25	2.25	1.5	1.5	0.75
m5C	Cm5C^WGG	ncog. tolerated	0.68	0.62	0.46	0.68	3.07	0.88	0.48	2.19	15.01	12.92	7.99	15.01	53.60	14.54	8.05	39.06	8	2.5	1.5	5.5	2.5	1.5	1.5	1
**NgoMIV (4ABT)**																										
m4C	G^Cm4CGGC	no data	1.89	1.89	0.13	0.63	5.87	4.20	0.18	1.68	24.13	24.13	1.45	10.39	82.87	56.70	1.71	26.17	7.5	4.5	1	3	2.5	1.5	1	1
m4C	G^CCGGm4C	no data	1.62	1.62	0.52	0.15	5.76	5.62	0.52	0.15	26.21	26.21	9.87	1.18	86.39	85.21	9.87	1.18	8	7	1	1	4	3	1	1
m5C	G^CCGGm5C	no data	1.38	1.38	0.63	0.13	3.84	3.61	0.63	0.23	22.42	22.42	13.58	1.12	60.77	58.96	13.58	1.81	5.5	4.5	1	1	2.5	2	1	0.5
m4C	G^m4CCGGC	no data	1.36	1.36	1.19	0.29	4.18	3.88	1.19	0.30	31.47	31.47	31.47	3.64	79.00	75.36	31.47	3.64	6.5	5	1	1.5	3.5	2	1	1.5
**m5C**	**G^m5CCGGC**	**cog. protective**	**1.25**	**1.25**	**0.80**	**0.99**	**5.82**	**3.78**	**0.80**	**2.04**	**26.72**	**23.77**	**19.24**	**25.31**	**110.29**	**68.28**	**19.24**	**42.01**	**8**	**4**	**1**	**4**	**2.5**	**1**	**1**	**1.5**
m5C	G^Cm5CGGC	ncog. protective	0.93	0.82	0.00	0.71	2.80	1.19	0.00	1.62	18.67	11.88	0.00	16.06	50.91	17.54	0.00	33.37	5	2	0	3	3	2	0	1
**PvuII (1PVI)**																										
m6A	Cm6AG^CTG	ncog. protective	1.86	1.86	0.38	0.02	5.05	5.03	0.45	0.02	23.53	23.53	6.29	0.03	72.25	72.23	6.73	0.03	8.5	8	1.5	0.5	3.5	3	1.5	0.5
m4C	m4CAG^CTG	no data	1.41	1.41	1.41	0.97	5.89	3.98	2.90	1.91	21.16	16.94	16.94	17.07	89.22	57.31	42.95	31.91	9.5	6.5	4	3	2	1	1	1
m5C	m5CAG^CTG	no data	0.72	0.72	0.54	0.69	3.55	1.49	0.54	2.06	15.65	13.55	10.63	15.34	60.93	23.29	10.63	37.64	7.5	3	1	4.5	2	1	1	1
**m4C**	**CAG^m4CTG**	**cog. protective**	**0.35**	**0.35**	**0.35**	**0.22**	**0.57**	**0.35**	**0.35**	**0.22**	**4.96**	**4.96**	**4.96**	**3.03**	**7.98**	**4.96**	**4.96**	**3.03**	**2**	**1**	**1**	**1**	**2**	**1**	**1**	**1**
m5C	CAG^m5CTG	no data	0.03	0.00	0.00	0.03	0.03	0.00	0.00	0.03	0.03	0.00	0.00	0.03	0.03	0.00	0.00	0.03	1	0	0	1	1	0	0	1
**ThaI (3NDH)**																										
**m4C**	**m4CG^CG**	**cog. protective**	**1.64**	**1.64**	**1.64**	**0.21**	**5.21**	**4.83**	**4.83**	**0.38**	**31.80**	**31.80**	**31.80**	**1.88**	**85.32**	**82.22**	**82.22**	**3.10**	**7**	**5**	**5**	**2**	**3**	**2**	**2**	**1**
m5C	CG^m5CG	no data	1.54	1.54	1.27	0.13	7.42	7.28	1.27	0.14	33.83	33.83	33.83	0.79	140.23	139.43	33.83	0.80	9	7.5	1	1.5	2	1	1	1
m5C	m5CG^CG	ncog. protective	1.36	1.36	1.36	0.00	6.46	6.46	3.61	0.00	28.15	28.15	28.15	0.00	114.02	114.02	65.57	0.00	8	8	4	0	2	2	1	0
m4C	CG^m4CG	no data	0.36	0.36	0.36	0.16	0.66	0.43	0.36	0.23	6.42	6.42	6.42	0.62	7.69	6.75	6.42	0.95	3.5	1.5	1	2	2	1	1	1
